# Influence of environmental conditions at spawning sites and migration routes on adaptive variation and population connectivity in Chinook salmon

**DOI:** 10.1002/ece3.8324

**Published:** 2021-11-16

**Authors:** Yara A. Alshwairikh, Shayla L. Kroeze, Jenny Olsson, Steve A. Stephens‐Cardenas, William L. Swain, Lisette P. Waits, Rebekah L. Horn, Shawn R. Narum, Travis Seaborn

**Affiliations:** ^1^ School of the Environment Yale University New Haven Connecticut USA; ^2^ Department of Biology Western University London Ontario Canada; ^3^ Department of Ecology and Environmental Science Umeå University Umeå Sweden; ^4^ Escuela de Ciencias Biológicas Universidad Latina de Costa Rica San Pedro Costa Rica; ^5^ Wildlife Genomics and Disease Laboratory Program in Ecology Department of Veterinary Sciences University of Wyoming Laramie Wyoming USA; ^6^ Department of Fish and Wildlife Sciences University of Idaho Moscow Idaho USA; ^7^ Columbia River Inter‐Tribal Fish Commission Hagerman Idaho USA

**Keywords:** genome–environment association, landscape genomics, local adaptation, migration, Pool‐Seq

## Abstract

Many species that undergo long breeding migrations, such as anadromous fishes, face highly heterogeneous environments along their migration corridors and at their spawning sites. These environmental challenges encountered at different life stages may act as strong selective pressures and drive local adaptation. However, the relative influence of environmental conditions along the migration corridor compared with the conditions at spawning sites on driving selection is still unknown. In this study, we performed genome–environment associations (GEA) to understand the relationship between landscape and environmental conditions driving selection in seven populations of the anadromous Chinook salmon (*Oncorhynchus tshawytscha*)—a species of important economic, social, cultural, and ecological value—in the Columbia River basin. We extracted environmental variables for the shared migration corridors and at distinct spawning sites for each population, and used a Pool‐seq approach to perform whole genome resequencing. Bayesian and univariate GEA tests with migration‐specific and spawning site‐specific environmental variables indicated many more candidate SNPs associated with environmental conditions at the migration corridor compared with spawning sites. Specifically, temperature, precipitation, terrain roughness, and elevation variables of the migration corridor were the most significant drivers of environmental selection. Additional analyses of neutral loci revealed two distinct clusters representing populations from different geographic regions of the drainage that also exhibit differences in adult migration timing (summer vs. fall). Tests for genomic regions under selection revealed a strong peak on chromosome 28, corresponding to the GREB1L/ROCK1 region that has been identified previously in salmonids as a region associated with adult migration timing. Our results show that environmental variation experienced throughout migration corridors imposed a greater selective pressure on Chinook salmon than environmental conditions at spawning sites.

## Graphical abstract

We assessed whether seven populations of ocean‐type lineage Chinook salmon in the Columbia River basin experience selection primarily driven by the migration corridor environment or by the spawning site environment. Results using whole‐genome resequencing suggest that environmental conditions at migration corridors may be more influential on Chinook salmon genetic variation compared with spawning site environmental conditions.

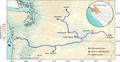

## 1 INTRODUCTION

Species that undergo extensive migrations often must move between habitats that are favorable and unfavorable at different life stages (Hecht et al., 2015; Lennox et al., 2016; Micheletti et al., 2018). These heterogeneous habitats may present unique selective pressures with varying strengths of selection. Specifically, migration‐driven challenges can act as selective pressures and be associated with adaptive variation in phenotypes within and among populations (Dingle, 1996). For example, some species’ migrations require persistent locomotion and movement beyond that of constitutive activity levels prior to or following migration (Wikelski et al., 2003). Some of these migratory species develop streamlined bodies that increase power and efficiency needed for traveling long and challenging migration routes (Ramenofsky & Wingfield, 2007). Extensive migration also leads to a redistribution of the population, which could result in isolated groups with reduced gene flow (Dingle & Drake, 2007).

Anadromous fish are migratory species that are born in freshwater, then migrate to the ocean where they remain for up to several years before returning to freshwater to spawn (Reist et al., 2006). Many researchers have evaluated if adaptive phenotypic variation in anadromous species is due to heterogeneity at breeding sites, migration corridors, or both. In the anadromous steelhead (*Oncorhynchus mykiss*), genetic adaptation was more closely related to the environment of migration corridors compared with the environment at spawning site (Micheletti et al., 2018). Additional studies found that timing of return migration and spawning are highly heritable traits in pink salmon (*O. gorbuscha*), Atlantic salmon (*Salmo salar*), and Chinook salmon (*O. tshawytscha*; Hansen & Jonsson, 1991; Quinn et al., 2000; Smoker et al., 1998).

The Chinook salmon is an anadromous species with an extensive migration and high philopatry. This species has considerable economic, social, cultural, and ecological value (Quaempts et al., 2018). Chinook salmon represent multiple genetic lineages and exhibit a wide range of life‐history variation in phenology of adult migration and sexual maturation (Quinn et al., 2015; Waples et al., 2004). In the interior Columbia River basin, there are two distinct Chinook lineages: ocean‐type and stream‐type (Healey, 1991; Narum et al., 2010). Ocean‐type Chinook salmon migrate to sea within a few months after hatching and do not return to freshwater until a few days or weeks before spawning. Stream‐type Chinook salmon remain in the river they were born in for much longer (over a year) before migrating to sea and return to freshwater much earlier in the year than ocean‐type Chinook salmon (Healey, 1991; Myers et al., 1998; Willis et al., 2021). These two lineages occur in sympatry in the Columbia River but are reproductively isolated due to local adaptation and isolation over geological time frames (Waples et al., 2004, 2008). Chinook are also classified into distinct maturation types based on their return migration timing. For the interior ocean‐type lineage, summer‐run refers to early returning fish that migrate to freshwater spawning grounds before they fully reach sexual maturity, while fall‐run refers to later returning individuals that wait to migrate until they are sexually mature (Quinn et al., 2015). Adult migration timing, which corresponds to phenotypes for early and late entry to freshwater and arrival to spawning grounds, is an important life‐history trait and has played a critical role in defining conservation units, such as Evolutionary Significant Units, which delineate distinct populations from one another (Waples & Lindley, 2018).

Extensive research has been done regarding genetic diversity and differentiation of Chinook salmon populations, including methods using restriction fragment length polymorphisms, mitochondrial DNA, allozymes, single nucleotide polymorphisms (SNPs), and microsatellites (Brannon et al., 2004; Hecht et al., 2015; Narum et al., 2008; Rasmussen et al., 2003; Waples et al., 2004). Results of these studies found a significantly higher amount of genetic divergence between lineages in the interior Columbia River basin compared with other regions throughout the species range. Moreover, there is considerable evidence of genetic associations with migration timing and spawning in this species (Hess et al., 2016; Koch & Narum, 2020; Narum et al., 2018; Prince et al., 2017; Thompson et al., 2019, 2020; Willis et al., 2021).

Previous landscape genomic studies of Chinook salmon identified local adaptation and potential environmental selective pressures. Population divergence was found to be associated with habitat variables such as temperature and elevation across life history types (Matala et al., 2011; Olsen et al., 2011). Additionally, a study using thousands of SNP markers from populations across the North American range of Chinook salmon found that 5.8–21.8% of population‐wide genomic variation can be accounted for by environmental features, the most significant being precipitation, temperature, and migration distance (Hecht et al., 2015). More recent whole‐genome sequencing work in Chinook salmon in the Columbia River has found extensive divergent selection throughout the genome, within and among genetic lineages. Narum et al. (2018) used association mapping with millions of genome‐wide SNPs and found that there was genetically determined phenotypic variation in adult migrating timing and arriving at spawning grounds in Chinook salmon in the interior lineages, which was consistent across phylogenetic lineages. However, the relative influence of environmental conditions along the migration corridor and at spawning sites on these genomic regions under selection is unknown.

In this study, we assessed whether seven populations of ocean‐type lineage Chinook salmon in the Columbia River basin experience selection primarily driven by the shared migration corridor environment or by the spawning site environment. Based on previous findings in anadromous steelhead (Micheletti et al., 2018), and because migration‐related challenges have been established as strong selective pressures (Dingle, 1996), we hypothesized that migration‐related environmental variables would have a stronger influence on selection compared with spawning site variables.

## 2 METHODS

### 2.1 Study area and sample collection

**FIGURE 1:**
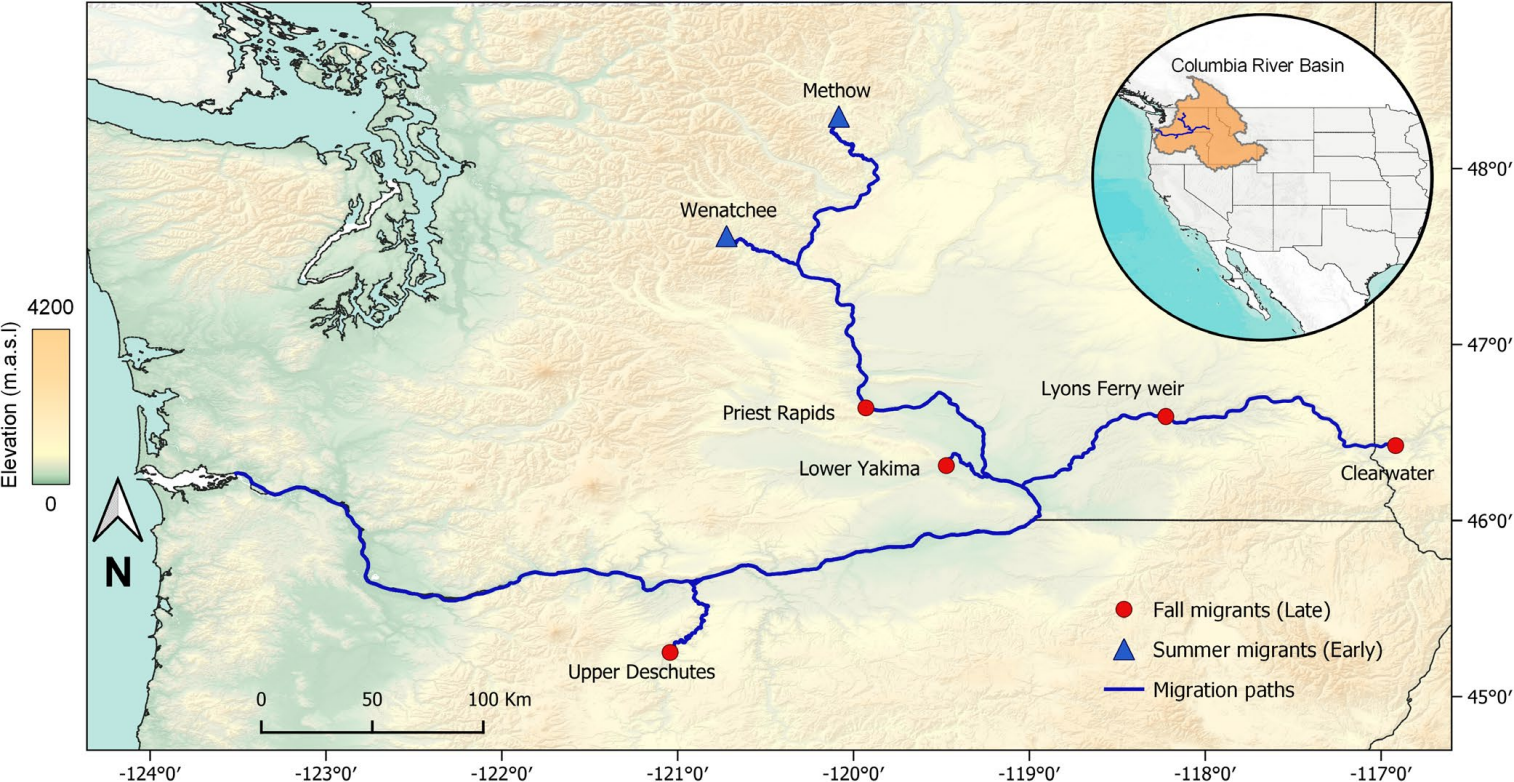
Sampling sites of ocean‐type Chinook salmon populations in the Columbia River basin. Fall‐run populations are indicated by red circles; Summer‐run populations are indicated by blue triangles. Base map imagery was obtained from the Esri database

The ocean‐type lineage of Chinook salmon is the predominant form in the southern freshwater range of the species, particularly in lower elevation and, coastal streams (Sharma & Quinn, 2012). Samples were collected from seven populations within this lineage that included two summer‐run and five fall‐run populations (Table 1; Figure 1). Juveniles were sampled from natural populations in the Methow River (*n* = 68) by a screw trap, and by beach seine in the Clearwater River (*n* = 96). We collected spawning adults from hatchery programs for Priest Rapids hatchery (*n* = 46), Yakima River (*n* = 46), Lyons Ferry hatchery (*n* = 92), and in traps placed in upstream ladders for Deschutes River (*n* = 48) and Wenatchee River (*n* = 61). Fin tissue samples were collected from each population as either outmigrating juveniles or returning adults in each river. Tissue samples were preserved in ethanol or dried on filter paper until DNA extraction. All fish in these Chinook salmon collections share a common portion of their migration corridors (Table A2; Figure 1). Inclusion of both natural and hatchery populations may introduce effects of hatchery and natural rearing on genomic variation. However, a study testing adaptive genomic variation of Chinook salmon across their North American range did not find rearing origin (hatchery vs. natural) to be a significant factor in their statistical models (Hecht et al., 2015). Therefore, we did not expect the use of both natural and hatchery populations to be a confounding factor in our study.

**TABLE 1:** Sample locations and overall descriptive features of Chinook fin tissue samples sequenced using an Illumina NextSeq

Population	Lat.	Long.	Migration season	Migration Phenotype	Number of samples	Number of reads	Mean coverage ± SD (filt)
Upper Deschutes River	45.250753	−121.043306	Fall	Late	48 (pool)	908,200,000	22.1 ± 12.1
Lower Yakima River	46.312190	−119.472570	Fall	Late	46	503,337,440	32.8 ± 15.8
Priest Rapids	46.640000	−119.930000	Fall	Late	46 (pool)	912,400,000	36.3 ± 16.6
Methow River	48.296000	−120.084000	Summer	Early	68	482,651,780	24.8 ± 13.0
Wenatchee River	47.616430	−120.722390	Summer	Early	61	476,295,447	32.0 ± 15.7
Clearwater River	46.426025	−116.917861	Fall	Late	96 (pool)	713,200,000	29.4 ± 14.1
Lyons Ferry weir	46.591330	−118.224830	Fall	Late	92	215,478,389	17.0 ± 9.3

Note

Geographical coordinates (Lat, Long), migration season, migration phenotype, and genetic sampling statistics are provided.

### 2.2 Landscape and environmental variables

For each population, we determined the migration route between each spawning site and the Pacific Ocean (the mouth of the Columbia River) by calculating the shortest path to the ocean using a stream network analysis along the national hydrology network developed by the USDA National Stream Internet project (Isaak et al., 2019). The estimated migration routes were confirmed by contrasting our results against previously known records of Chinook salmon distributions and activity for both summer/fall seasons, provided by the Pacific States Marine Fisheries Commission (www.streamnet.org). We also calculated the percent overlap of migration routes among all seven populations of Chinook salmon, by estimating the pairwise ratio of the overlap distance to the distance of the smallest path (Barraclough & Vogler, 2000). This index, transformed to percent, ranges from 0% (no overlap) to 100% (complete overlap with respect to the population with the shorter path).

A 5‐km buffer around each of the seven spawning sites was used to establish an area of environmental and topographic influence and account for variation at those sites (Micheletti et al., 2018; Micheletti & Storfer, 2017). For migration routes, environmental and topographic descriptors were extracted using a 500‐m buffer for segments along each river line included in the migration route. Both analyses were performed using the ArcGIS Pro v.2.4.0 (ESRI) Spatial Analysis toolbox and buffer tools, respectively. These two steps created the geographic locations where we extracted environmental variables: at the spawning site, or along the migration route.

Environmental variables, describing both bioclimatic and topographic conditions at the spawning site and the migration route, were collected from open source GIS databases. We included environmental predictors that are known to be associated with genetic variation of salmon (Hecht et al., 2015; Matala et al., 2011; Micheletti et al., 2018), and additional topographic and environmental descriptor variables of the study area (Table 2). We first collected bioclimatic variables representing annual trends and environmental seasonality (19 BIOCLIM), in addition to average wind speed and solar radiation data, from WorldClim V2.0 (Fick & Hijmans, 2017), at a ~1 km^2^ resolution. We obtained the SRTM digital elevation model of the Columbia River basin (~30 m^2^) from USGS (earthexplorer.usgs.gov). Tree Canopy Cover (2016 TCC) percentage was collected by the USDA Forest Service, and stream temperature and stream slope data were obtained from the NorWest database (Isaak et al., 2011) at the highest resolution available. The number of dams along the migration routes was gathered from the Global Reservoir and Dam Database provided by NASA‐SEDAC (Lehner et al., 2011). Additional topographic‐based variables—Heat Load Index, terrain roughness, migration distance and stream order—were derived from the Elevation model. For migration routes, we extracted descriptive values from raster data and calculated the mean, minimum, maximum, and range statistics for each variable because migration routes span a large geographic range (Micheletti et al., 2018). Whereas for the spawning sites, we only calculated the mean values for each variable within the 5‐km buffer as the descriptor of the variation in environmental and topographic conditions present at each location. The complete GIS management, data extraction and analysis were performed using ArcGIS Pro v.2.4.0 (ESRI) and R statistical software (R Core Team, 2020).

**TABLE 2:** List of the environmental variables used in GEA analyses

Environmental variable	Environmental variable code	Statistic	Location	GEA test	Dataset	Source	Geographic resolution
Annual mean temperature	Bio1_mean	Mean	Migration corridor	AutoLM, RDA, LFMM, BayPass	Full, Migration	Worldclim	1 km^2^
Annual mean temperature	Bio1_site	Mean	Spawning site	AutoLM, LFMM, BayPass	Full	Worldclim	1 km^2^
August water temperature (20‐year mean)	RvT_range	Range	Migration corridor	AutoLM, RDA, LFMM, BayPass	Full, Migration	Norwest	1 km^2^
August water temperature (20‐year mean)	RvT_site	Mean	Spawning site	AutoLM, LFMM, BayPass	Full	Norwest	1 km^2^
August water temperature (20‐year mean)	RvT_mean	Mean	Migration corridor	AutoLM, LFMM, BayPass	Full	Norwest	1 km^2^
Isothermality (mean annual temperature/annual temperature range) (*100)	Bio3_site	Mean	Spawning site	AutoLM, RDA, LFMM, BayPass	Full, Combined, Site	Worldclim	1 km^2^
Isothermality (mean annual temperature/annual temperature range) (*100)	Bio3_mean	Mean	Migration corridor	AutoLM, LFMM, BayPass	Full	Worldclim	1 km^2^
Max temperature of warmest month	Bio5_max	Maximum	Migration corridor	AutoLM, LFMM, BayPass	Full	Worldclim	1 km^2^
Max temperature of warmest month	Bio5_mean	Mean	Migration corridor	AutoLM, LFMM, BayPass	Full	Worldclim	1 km^2^
Max temperature of warmest month	Bio5_range	Range	Migration corridor	AutoLM, RDA, LFMM, BayPass	Full, Combined, Migration	Worldclim	1 km^2^
Max temperature of warmest month	Bio5_site	Mean	Spawning site	AutoLM, RDA, LFMM, BayPass	Full, Combined, Site	Worldclim	1 km^2^
Mean diurnal temperature range	Bio2_mean	Mean	Migration corridor	AutoLM, LFMM, BayPass	Full	Worldclim	1 km^2^
Mean diurnal temperature range	Bio2_site	Mean	Spawning site	AutoLM, LFMM, BayPass	Full	Worldclim	1 km^2^
Mean temperature of coldest quarter	Bio11_site	Mean	Spawning site	AutoLM, LFMM, BayPass	Site	Worldclim	1 km^2^
Mean temperature of warmest quarter	Bio10_range	Range	Migration corridor	AutoLM, RDA, LFMM, BayPass	Combined	Worldclim	1 km^2^
Temperature seasonality	Bio4_site	Mean	Spawning site	AutoLM, LFMM, BayPass	Full	Worldclim	1 km^2^
Temperature seasonality (standard deviation * 100)	Bio4_max	Maximum	Migration corridor	AutoLM, LFMM, BayPass	Full	Worldclim	1 km^2^
Temperature seasonality (standard deviation * 100)	Bio4_mean	Mean	Migration corridor	AutoLM, LFMM, BayPass	Full	Worldclim	1 km^2^
Temperature seasonality (standard deviation * 100)	Bio4_range	Range	Migration corridor	AutoLM, LFMM, BayPass	Full	Worldclim	1 km^2^
Annual precipitation	Bio12_mean	Mean	Migration corridor	AutoLM, LFMM, BayPass	Full	Worldclim	1 km^2^
Annual precipitation	Bio12_min	Minimum	Migration corridor	AutoLM, LFMM, BayPass	Full	Worldclim	1 km^2^
Annual precipitation	Bio12_range	Range	Migration corridor	AutoLM, LFMM, BayPass	Full	Worldclim	1 km^2^
Precipitation of driest month	Bio14_mean	Mean	Migration corridor	AutoLM, LFMM, BayPass	Full	Worldclim	1 km^2^
Precipitation of wettest month	Bio13_mean	Mean	Migration corridor	AutoLM, LFMM, BayPass	Full	Worldclim	1 km^2^
Precipitation of wettest month	Bio13_min	Minimum	Migration corridor	AutoLM, LFMM, BayPass	Full	Worldclim	1 km^2^
Precipitation of wettest month	Bio13_range	Range	Migration corridor	AutoLM, LFMM, BayPass	Full	Worldclim	1 km^2^
Precipitation seasonality (coefficient of variation)	Bio15_site	Mean	Spawning site	AutoLM, RDA, LFMM, BayPass	Full, Site	Worldclim	1 km^2^
Precipitation seasonality (coefficient of variation)	Bio15_mean	Mean	Migration corridor	AutoLM, RDA, LFMM, BayPass	Full, Migration	Worldclim	1 km^2^
Precipitation seasonality (coefficient of variation)	Bio15_min	Minimum	Migration corridor	AutoLM, LFMM, BayPass	Full	Worldclim	1 km^2^
Precipitation seasonality (coefficient of variation)	Bio15_range	Range	Migration corridor	AutoLM, LFMM, BayPass	Full	Worldclim	1 km^2^
Elevation	DEM_max	Maximum	Migration corridor	AutoLM, LFMM, BayPass	Full	USGS	30 m^2^
Elevation	DEM_mean	Mean	Migration corridor	AutoLM, LFMM, BayPass	Full, Migration	USGS	30 m^2^
Elevation	DEM_range	Range	Migration corridor	AutoLM, LFMM, BayPass	Full, Migration	USGS	30 m^2^
Elevation	DEM_site	Mean	Spawning site	AutoLM, LFMM, BayPass	Full	USGS	30 m^2^
Elevation roughness	Rough_mean	Mean	Migration corridor	AutoLM, LFMM, BayPass	Migration	Calculated in ArcGis	30 m^2^
Elevation roughness	Rough_site	Mean	Spawning site	AutoLM, LFMM, BayPass	Site	Calculated in ArcGis	30 m^2^
Slope	Slp_mean	Mean	Migration corridor	AutoLM, LFMM, BayPass	Full, Combined	Norwest	1 km^2^
Heat Load Index	HLI_site	Mean	Spawning site	AutoLM, RDA, LFMM, BayPass	Combined, Site	Calculated in ArcGis	30 m^2^
Solar radiation	SRad_mean	Mean	Migration corridor	AutoLM, LFMM, BayPass	Combined	Norwest	1 km^2^
Wind velocity	Wsp_range	Range	Migration corridor	AutoLM, LFMM, BayPass	Combined	Worldclim	1 km^2^
Wind velocity	Wsp_site	Mean	Spawning site	AutoLM, RDA, LFMM, BayPass	Full, Combined, Site	Worldclim	1 km^2^
Migration distance to ocean	mig_distance	Sum	Migration corridor	AutoLM, RDA, LFMM, BayPass	Migration	Calculated in ArcGis	1 km^2^
Number of dams	Dam_Num	Sum	Migration corridor	AutoLM, LFMM, BayPass	Full	NASA ‐ Sedac	1 km^2^
Stream order	stOrd_mean	Mean	Migration corridor	AutoLM, RDA, LFMM, BayPass	Full, Combined	Calculated in ArcGis	30 m^2^
Stream order	stOrd_site	Mean	Spawning site	AutoLM, LFMM, BayPass	Combined	Calculated in ArcGis	30 m^2^

Note

For the migration route variables, mean, minimum, maximum, and range statistics were extracted. For spawning sites (all variables but number of dams and migration distance), an average of the value in the 5‐km buffer was extracted. For discrete variables such as dam number along migration route and stream order, we estimated only the total number of dams and average stream hierarchy along the path, respectively. The “GEA test” column indicates the tests that a given environmental variable was used in. The “Dataset” column indicates which of the three datasets, “Full,” “Migration,” “Site,” and “Combined” that the environmental variable was included in.

These steps created a final dataset of 140 landscape‐derived variables across spawning sites and migration corridors. A total of 98 out of the 140 variables showed variation in at least one spawning site or migration corridor. To reduce multicollinearity for downstream analyses, we created several subset datasets by taking an iterative approach of manual and automated removal of variables. Ignoring Spearman rank correlations where *p* > .05, we performed a 4‐step manual stepwise removal of variables that were most correlated based on the *R* ^2^ value (*R* ^2^ > 0.80), followed by automated removal of variables with *R* ^2^ > 0.80 using the psych and caret packages in R. This left us with 10 variables. We manually added back in variables of biological importance for Chinook salmon (Micheletti et al., 2018) or other salmonids in the area (e.g., bio 3 and bio 5, distance along migration corridors), and then proceeded to manually remove variables highly correlated with those target variables. The full code and specific filtering steps can be found on Github: https://github.com/YaraAlshw/LG_Chinook. The matrix for the 140 variables is available on Dryad: https://doi.org/10.5061/dryad.prr4xgxmn. These steps created three datasets: The dataset “Combined” contained a total of 11 variables reflecting a mix of both migration and spawning site variables; the second dataset, “Migration” contained eight variables that are exclusively migration‐specific; and the third dataset “Site” contained seven variables that are exclusively site‐specific (Table 2).

The three datasets, “Combined,” “Migration,” and “Site” were used for analyses where multiple environmental factors were tested concurrently. For downstream analyses where variables were tested independently (i.e., latent factor mixed‐modeling (LFMM); see below), we created an additional dataset using a less strict elimination method based on percentage of correlation to other variables in order to retain a higher number of variables. Starting with the set of 98 variables, we removed highly correlated variables (*R* ^*2*^ > 0.80) only if they were correlated to >50% of the variables within the dataset. This process allowed us to retain a set of 36 environmental variables becoming the fourth environmental dataset named “Full” (Table 2).

We used the “Migration” and “Site” datasets to implement a preliminary Neighbor‐Joining analysis with Euclidean distances to test environmental similarity between each of the geographical locations sampled, and between each of the migration paths connecting these locations with the ocean. This analysis was implemented using the *vegan* package in R using the site location variables or the migration variables (Oksanen et al., 2019; R Core Team, 2020).

### 2.3 Genomic sequencing

We followed a PCR‐based Chelex method for DNA extraction as per Sweet et al. (1996), with modifications including pre‐extraction preparation with proteinase, incubations at 56 and 100 °C, and microconcentration of the solution. We used a pooled‐sequencing approach for whole genome re‐sequencing (Schlötterer et al., 2014). Specific methods for pooling of samples and library preparation followed that of Narum et al. (2018) for the Deschutes (*n* = 48), Priest Rapids (*n* = 46), and Clearwater River (*n* = 96) samples. For the lower Yakima River (*n* = 46), Methow River (*n* = 68), Wenatchee River (*n* = 61), and Lyons Ferry (*n* = 92) samples, individual samples were barcoded with adapters using the NEBNext Ultra Kit before samples were pooled for sequencing. For a detailed description on the methods for individually barcoding pooled samples for sequencing, see Horn et al. (2020). All sequencing was performed on an Illumina NextSeq500.

Genomic data were processed using the PoolParty v0.8 pipeline, a bioinformatics resource for pooled sequencing data that integrates several data processing tools, including PoPoolation2 (Kofler et al., 2011) into a single pipeline (Micheletti & Narum, 2018). PoolParty utilizes BBduk from BBMap v38 (Bushnell, 2016) to trim reads with a base quality score of 20 and a minimum length of 25. Trimmed reads were aligned to the reference GenBank assembly of *O. tshawytscha* (GCA_002872995.1) using BWA‐MEM (Li, 2013) with default parameters and a minimum mapping quality score of 5. PCR duplicates were identified and removed using SAMBLASTER (Faust & Hall, 2014). Finally, BAM files were filtered and sorted using toolkit Picard v2.0.1 (Picard Toolkit, 2019) and SAMtools (Li, 2011). SNP positions were called with BCFtools using a minimum SNP quality of 20 and an indel window of 10. SNPs with a minor allele frequency (MAF) below 0.05 and a depth of coverage below 10 were removed. All analyses hereafter are based on allele frequencies and not hard‐called SNPs. The final data set is summarized in Table 1.

### 2.4 Neutral population genetic structure

We examined neutral population genetic structure with a subset of SNPs that were filtered to remove putatively adaptive regions using the “AFFILT” allele frequency filter set to 0.1 (difference in allele frequencies) in the PPanalyze module of PoolParty. SNPs were further filtered to include only those with a minimum and maximum coverage of 15 and 250, respectively. Minimum coverage filters were necessary to achieve adequate read depth to estimate allele frequencies, while maximum coverage filters were intended to reduce SNPs from homologous regions of the genome. Using the PPanalyze module, we constructed a Neighbor‐Joining tree and principal components analysis (PCA) to visualize the neutral population genetic structure present among ocean‐type lineage populations. The SYNC file created by PPanalyze was used to calculate global and pairwise FST values using the R package *poolfstat* v1.1.1 (Hivert et al., 2018) to quantify the amount of population genetic structure among populations. A set of custom bash and R scripts were used to calculate confidence intervals for each FST value (Dorant et al., 2019). A Mantel test was used to identify correlation between neutral genetic differentiation (pairwise FST values) and pairwise geographic overlap among populations, using the *vegan* package in R.

### 2.5 Identifying genomic regions under selection

There are many genome‐wide association tests to detect genomic regions possibly under selection with varying rates of false‐positives and false‐negatives, each with their own test assumptions. We performed several genome‐wide association tests using the PPanalyze module of PoolParty, and a Bayesian approach that is independent of the PoolParty pipeline. These outlier tests included FST and sliding window FST tests (SFST; Karlsson et al., 2007), Fisher's exact test (FET), and an extended Lewontin and Krakauer test (FLK; Bonhomme et al., 2010). In FST tests, the overall distribution and variance of FST values are used to identify loci under selection (Beaumont & Nichols, 1996; Flangan & Jones, 2017; Lewontin & Krakauer, 1973). Whereas the sliding window FST test calculates FST values at specific genomic regions. The FLK test takes into account the hierarchical structuring of populations unlike traditional FST tests. In FET, SNPs are tested for differences in allele frequencies. For all analyses, SNP positions were filtered to exclude those with coverage below 15× and greater than 250×. In order to categorize genomic regions under selection while minimizing the underlying effects of genetic structure, four runs of PPanalyze were undertaken to screen for significant SNPs that were (1) among all populations, (2) between the combined summer‐ and combined fall‐run populations, (3) among the summer‐run populations, and (4) among the fall‐run populations.

For a Bayesian approach, we used the program BayeScan v2.1 (Foll & Gaggiotti, 2008) to screen for SNPs located in regions under selection. Specifically, this test provides an estimate of the posterior probability that a locus is under selection (Foll & Gaggiotti, 2008). We converted the SYNC file into a GenePop file format using the PoPoolation2 command “subsample_sync2GenePop” with a minimum allele count of four and a minimum and maximum coverage of 40 and 250, respectively. These settings allowed us to simulate 40 “genotyped” individuals to be used for the analysis. A custom set of scripts were used to merge chromosome and scaffold level genepop files into one (https://github.com/esnielsen/MSc‐bioinformatics). An R script (Ravinet, 2014) was used to convert the genepop file to a BayeScan input file. BayeScan was run using the SNP matrix option and a prior odds of 100.

### 2.6 Genome–environment association analyses

We used several approaches to test for genome–environment associations (GEA), using one or more of the four environmental datasets we identified earlier. Tests for GEA were completed in four different analyses packages: redundancy analysis (RDA), AutoLM, LFMM, and BayPass. Each of these tests has different model assumptions and offers benefits and drawbacks. Univariate approaches such as AutoLM, LFMM, and BayPass test a single locus and a single variable (Forester et al., 2018). These approaches offer a comprehensive testing design and are expected to not miss important adaptive loci or predictors (Rellstab et al., 2015), but risk increasing the rate of false positives (Forester et al., 2018). Additionally, the issue of “interdependent models” is introduced when environmental predictors are highly correlated (Rellstab et al., 2015). Multivariate approaches can overcome the issue of multicollinearity through the use of PCA to summarize the contribution of environmental variables into a synthetic environmental variable (Micheletti et al., 2018). However, this can make the interpretation of results difficult.

We first performed a set of RDA tests using the R package vegan v 2.5‐6 (Oksanen et al., 2019). RDA represents a multivariate linear regression approach to GEA analyses (Forester et al., 2018). For frequentist univariate approaches to GEA, we implemented both AutoLM and LFMM analyses. AutoLM is a linear mixed‐effects model that can detect significant associations between allele frequencies and environmental variables and incorporates spatial autocorrelation as a covariate (Micheletti et al., 2018). We performed four sets of AutoLM analysis using all four environmental datasets described earlier using the source code for AutoLM (https://github.com/StevenMicheletti/autoLM). We used the function lfmm2 from the source code (https://rdrr.io/bioc/LEA/src/R/lfmm2.R) to perform the LFMM analysis, which uses an exact least‐squares approach to estimate latent factors and ultimately identifies candidate SNPs that associate with an environmental variable (Caye et al., 2019). We set the value of *K* = 2 based on NGSadmix analysis (Skotte et al., 2013). Briefly, the input file for NGSadmix was created using the ANGSD program (Korneliussen et al., 2014) to convert the filtered mpileup file into genotype likelihoods (for the pooled populations). NGSadmix was run with a *K* value ranging from 1 to 8, with 10 iterations of each *K* value to assess proper convergence and the most likely *K* was identified based on the likelihood scores using the Clumpak server (Kopelman et al., 2015; Figure S1). Analyzing pooled sequencing data using LFMM risks loss of power, and to avoid this issue we simulated “genotypes” for 20 individuals per population using the function rbeta in R, based on the recommendations from the LFMM FAQ page and personal communication with the authors (https://membres‐timc.imag.fr/Olivier.Francois/lfmm/faq.htm; O. Francois 2020, personal communication Jul 21) and expanded the environmental dataset input files to 20 identical observations per population to match the simulated genotypes input file. Our analysis strategy differed from the standard LFMM analysis which entails creating a PCA on the environmental variables and using the first axis as the input for the environmental data. We were interested in identifying and interpreting the contribution of each environmental variable separately. Therefore, we performed each lfmm2 iteration independently using each unique variable from the four environmental datasets: “Site,” “Migration,” “Combined,” and “Full” dataset.

To correct for multiple testing in both AutoLM and LFMM analyses, we calculated Bonferroni corrections and Benjamini & Hochberg (BH) correction factors. We manually calculated the Bonferroni correction as alpha of 0.05 divided by the total number of SNPs. For the BH correction, we used the IHW v 1.15.0 R package (Ignatiadis et al., 2016). We plotted raw *p*‐values using Manhattan plots to visualize significant associations and look for genomic regions that showed large peaks. We filtered for candidate SNPs that had *p*‐values below the threshold identified by the Bonferroni approach because it is more conservative than the BH approach.

Lastly, for a univariate Bayesian approach to GEA, we used the program BayPass (Gautier, 2015) to describe adaptive differences across the genome. The SYNC file was used to convert to a BayPass input file using the Poolfstat R package (Hivert et al., 2018). Four different runs of BayPass were performed that correlated with the four environmental datasets. Each covariate file was standardized by BayPass before analysis. Bayes factor (BF) values across the genome were plotted using Manhattan plots to screen for genomic regions that may be highly associated with each environmental variable. BF values (BF ≥ 20; Jarosz & Wiley, 2014) were extracted from the betai output file for comparison across GEA and outlier tests.

To consolidate the results from the GEA tests and produce a set of candidate SNPs for gene ontology (GO) investigation, we filtered for SNPs that were identified as significant by at least one BayPass test, and at least one of the multivariate or frequentist univariate tests. We then used the package SNP2GO (Szkiba et al., 2014) to test for enrichment of those SNPS. First, we used VCFtools (Danecek et al., 2011) to generate VCF files of the candidate SNPs and all non‐candidate SNPs. We also tested for enrichment of SNPs that were in common between at least one outlier test and one GEA test. We used the *O*. *mykiss* genome annotation (v100; Yates et al., 2020) because the annotated Chinook salmon genome lacked the “Gene ontology” term needed for the SNP2GO analysis.

## 3 RESULTS

### 3.1 Landscape and environmental variables

**FIGURE 2:**
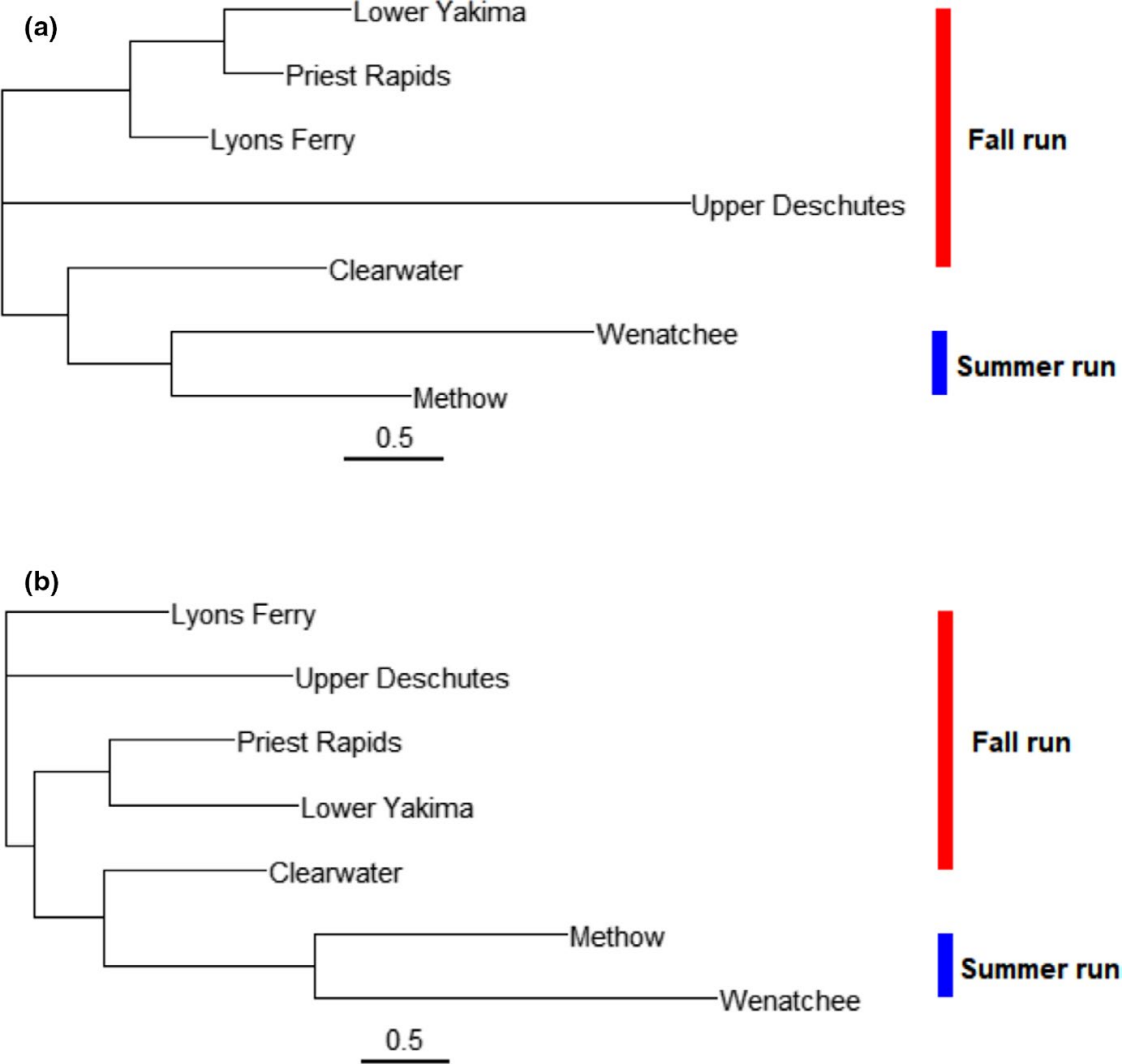
Neighbor‐joining tree of environmental data for (a) spawning site locations and (b) migration paths for seven populations of Chinook salmon

Cluster analysis showed that geographical locations sustaining populations with the same migration timing tend to be more environmentally similar to each other than to other locations inhabited by populations migrating at different life stages (Figure 2). Pairwise comparisons of percent overlap of migration routes indicated that, as part of the main river basin, each population shares large river sections with each other, ranging from 65% to 100% overlap with respect to the population with the shorter path (Table S2). Although the overlap index does not account for symmetric overlapping, only three pairwise comparisons of migrations river lines showed complete overlap. The Lyons Ferry migration path completely overlaps with a large section of the Clearwater path, and the fall‐run Priest Rapids migration river line was completely overlapped with the two summer‐run population river lines, Wenatchee and Methow.

### 3.2 Genomic data

The number of reads per pooled library ranged from almost 216 million to over 900 million (Table 1). After filtering steps, the average coverage across the genome ranged from 17× to 36× (Table 1). There were a total of 64,966,505 SNPs called before filtering with 36,371,688 removed due to a quality score less than 20 and total depth of position less than 10. An additional 15,492,375 SNPs were removed after applying a MAF of 0.05. A total of 13,102,442 variants remained after filtering for analysis, with 10,823,935 being SNPs and 2,278,507 as indels.

### 3.3 Neutral population genetic structure

**FIGURE 3:**
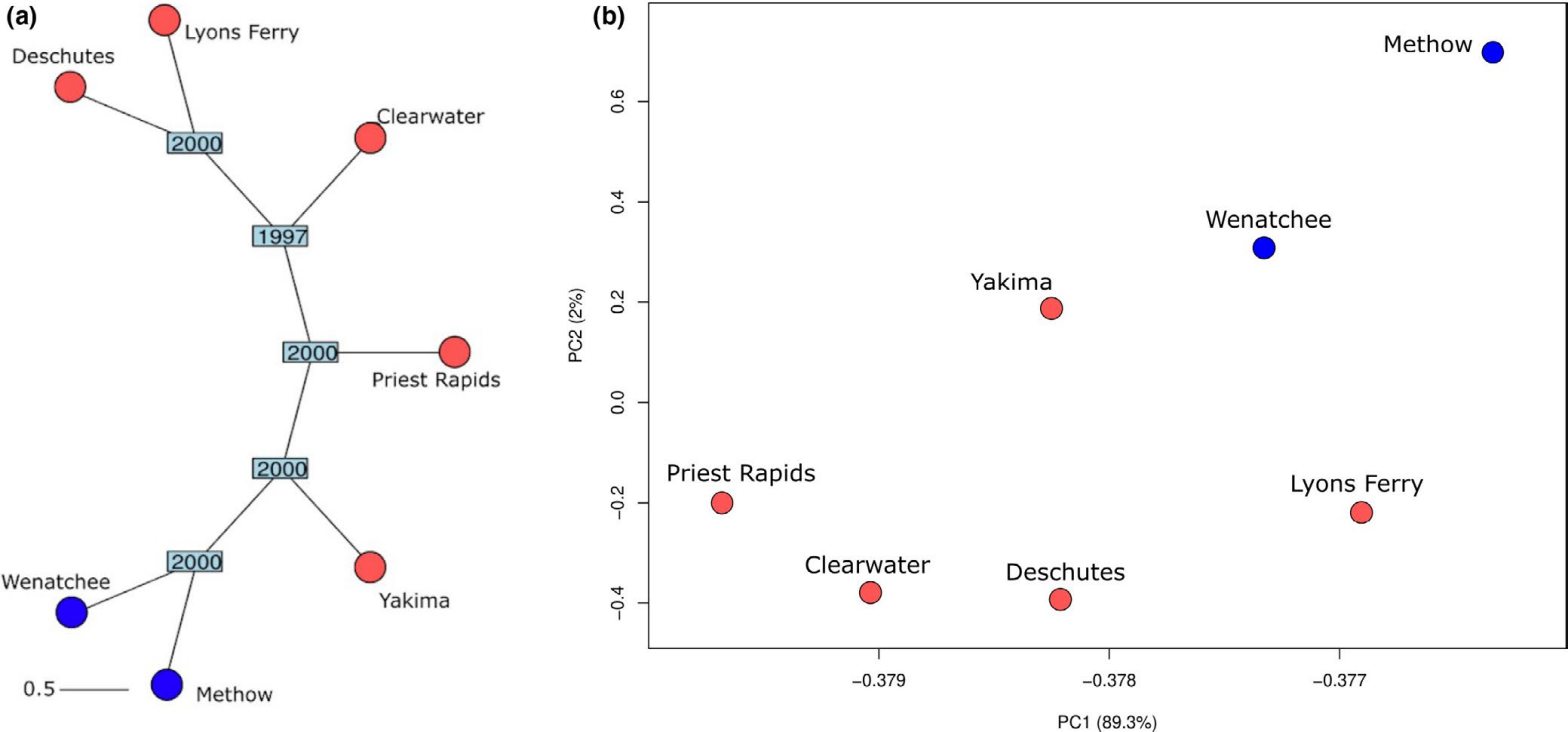
(a) Consensus Neighbor‐Joining tree of all populations of Chinook salmon using the filtered set of putatively neutral SNPs (4,212,127). Blue nodes indicate summer‐run populations, and red nodes indicate fall‐run populations. Bootstrap support from 2000 iterations is shown in boxes. In (b) PCA of all populations using the filtered set of putatively neutral SNPs (4,212,127). Blue circles indicate summer‐run populations, and red circles indicate fall‐run populations

**FIGURE 4:**
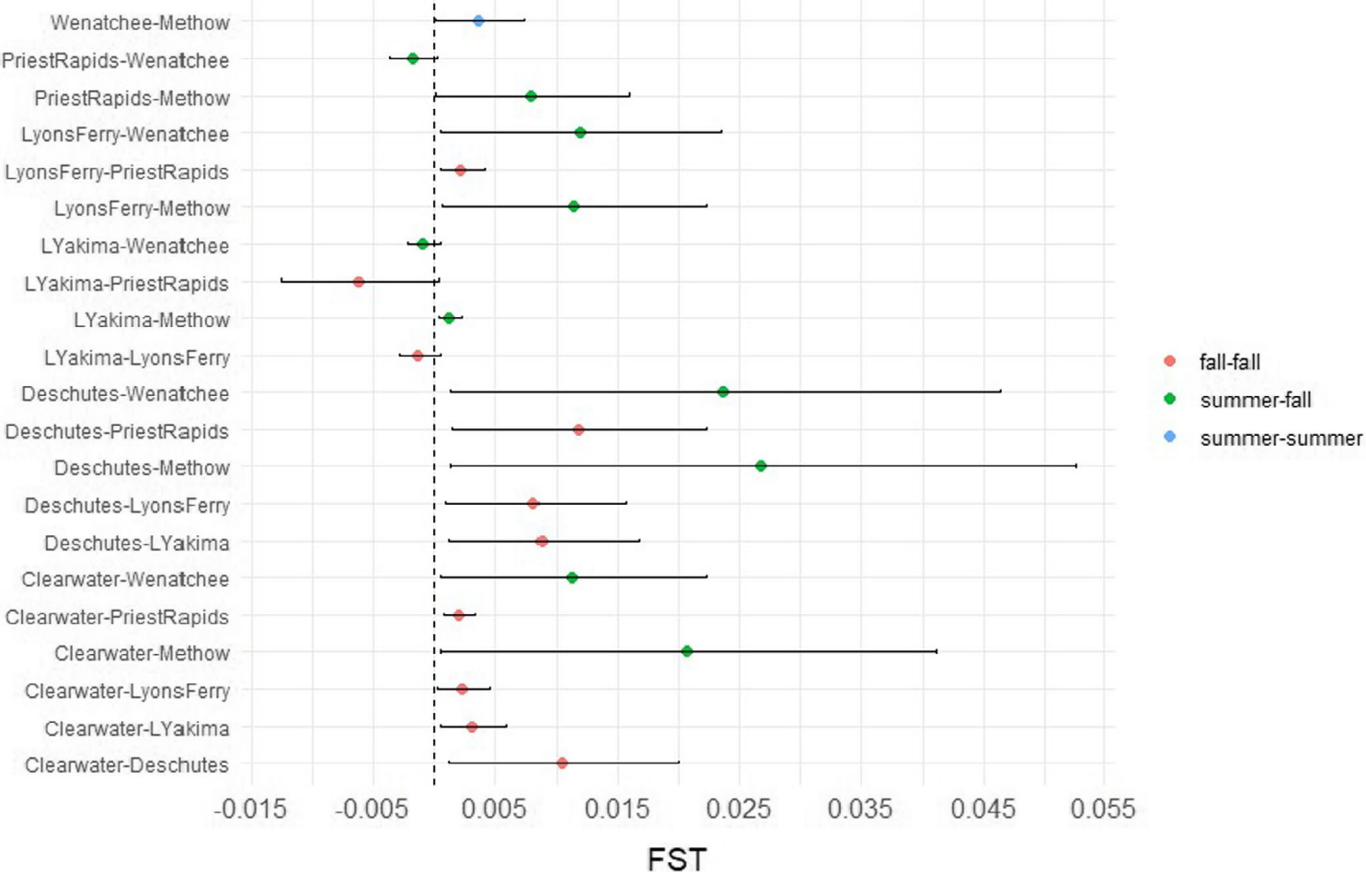
Pairwise FST values (represented as circles) for pairs of Chinook salmon based on putatively neutral SNPs and colored by return migration season. The horizontal lines for each pairwise comparison represent the confidence intervals

A total of 4,212,127 sites remained after filtering for putatively neutral SNPs. Both the NJ tree (Figure 3a) and PCA (Figure 3b) indicated clustering of the summer‐run populations separately from the fall‐run populations. In the PCA, the first axis explained the majority of the variation (89.3%) compared with axis two (2%; Figure 3b). The overall FST was 0.007, with pairwise FST values ranging from 0 to 0.027 (Table S1). The confidence intervals (CI) for four pairwise comparisons (Table S1) overlapped zero, indicating no genetic structure among these populations. The average pairwise FST value in comparisons among fall‐run populations was 0.006, whereas the average pairwise FST value in comparisons of fall‐ to summer‐run populations was 0.014, although confidence intervals were overlapping (Figure 4). The largest pairwise comparisons are among the summer‐run populations (Wenatchee and Methow) and the fall‐run Deschutes River population (FST = 0.024 and 0.027, respectively). The Mantel test showed significant negative correlation (Mantel *r* = −.644, *p* = .002) between neutral genetic structure and geographic overlap on migration paths. Populations with highly overlapped migration corridors presented low neutral genetic structure despite different migratory timing.

### 3.4 Identifying genomic regions under selection

The FST, SFST, FET, and FLK tests screening for genomic regions under selection among all populations revealed several regions across the genome in common among tests (Figure S2a–d). The most notable peak was on Chr28, and corresponds to the GREB1L/ROCK1 region. Another region identified by all tests is located on Chr19 (Figure S2a–d) and corresponds to a ladderlectin‐like gene. Ladderlectin in rainbow trout (*O*. *mykiss*) has been shown to be involved in the immune response for bacterial and fungal pathogens (Reid et al., 2011). In addition, the FLK, FST, and SFST test identified regions on Chr10, Chr12, Chr13, Chr14, and Chr17 (Figure S2a,b,d). The region on Chr10 contains several genes and the intergenic space. On Chr12 the SNPs are mostly located in intergenic regions, but also on a gene called MLX‐interacting protein, a transcription activation protein (Singh & Irwin, 2016). The peak on Chr13 corresponds to SNPs in the gene H‐2 class I histocompatibility antigen, in which genes belonging to the MHC family are involved in immune response. The Chr14 peak contains SNPs that are located in the gene named CMP‐N‐acetylneuraminate‐beta‐galactosamide‐alpha‐2,3‐sialyltransferase 4‐like that is involved in protein modification pathways (Uniprot Q11201). Lastly, the SNPs underlying the peak on Chr17 lie in intergenic regions and the gene calcium/calmodulin‐dependent protein kinase type 1D (CAM kinase 1D) in the SFST test. CAM kinase 1D is involved in the calcium signaling cascade and acts as an activator (Uniprot Q8IU85).

**FIGURE 5:**
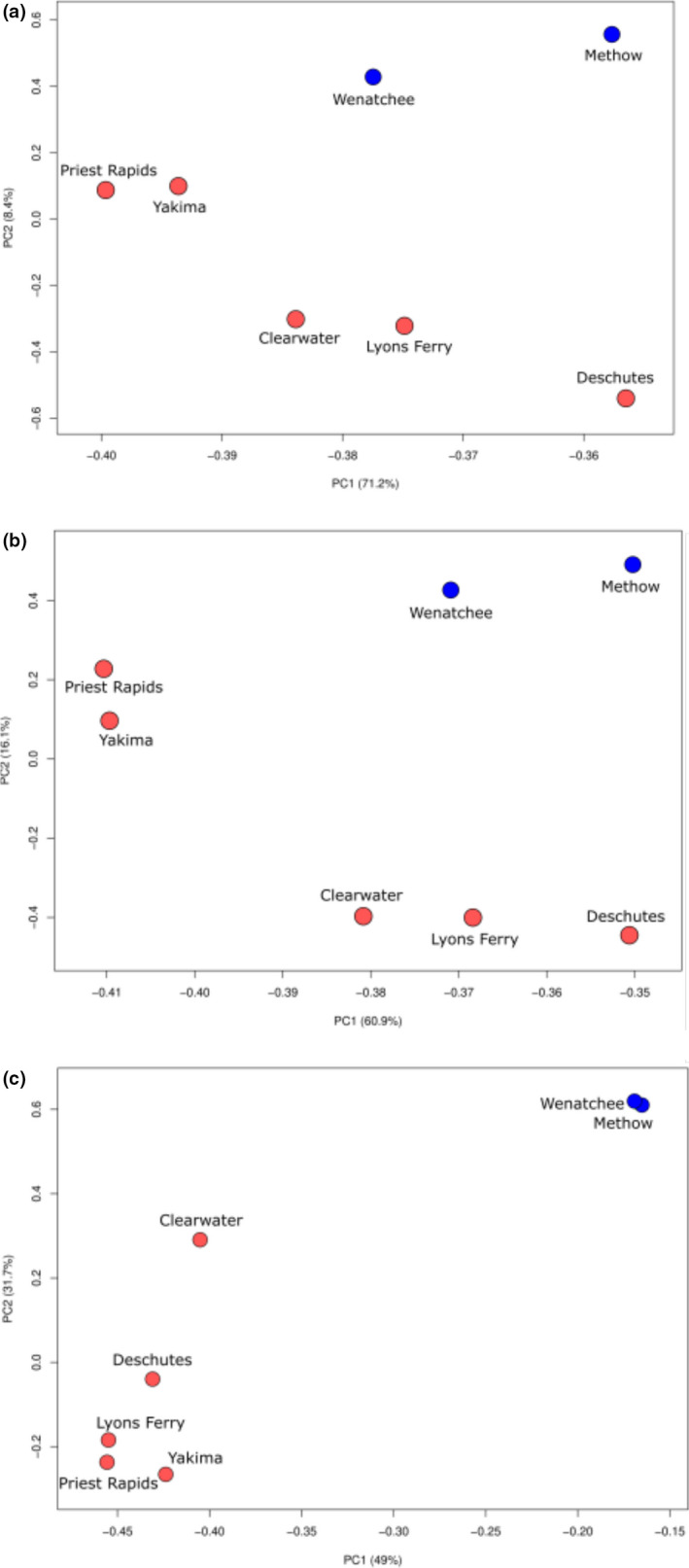
Principal component plot comparing the summer‐ (blue) and fall‐ (red) run populations of Chinook salmon using (a) the full, filtered genomic dataset, (b) the SNP positions determined by the sliding FST test on Chr2, Chr9, Chr10, Chr12, Chr13, Chr14, Chr17, Chr19, and Chr29, and (c) the SNP positions determined by the sliding FST test on Chr28

In the analysis screening for putatively adaptive SNPs between summer‐ and fall‐run fish, the largest association is again located on Chr28 in the GREB1L/ROCK1 region (Narum et al., 2018). All tests identified a significant region on Chr19, which like the previous tests performed among all populations, corresponds to a ladderlectin‐like gene. The peaks observed on Chr10, Chr12, Chr13, and Chr14 in the FST, SFST, and FLK tests (Figure S3a,b,d) are congruent with the peak observed in the previous analysis, implying that the significant SNPs regions observed between summer‐ and fall‐run fish are likely driving the results in the previous analysis (among all populations). The SNPs underlying the peaks on Chr2, Chr9, Chr10, Chr12, Chr13, Chr14, Chr17, Chr19, Chr28, and Chr29 from the sliding FST test were extracted from the SYNC file. PPanalyze was used to run a PCA using just the SNP positions underlying the peak on Chr28 and again using the SNP positions from Chr2, Chr9, Chr10, Chr12, Chr13, Chr14, Chr17, Chr19, and Chr29. These PCA plots were compared with the PCA using all filtered, genomic positions to determine the extent of population differentiation based on seasonal run‐timing (Figure 5). The PCA using all SNP positions was generally identical to the PCA using only those SNPs found on Chr2, Chr9, Chr10, Chr12, Chr13, Chr14, Chr17, Chr19, and Chr29 (Figure 5a,b). The PCA using SNPs from Chr28 only produced the greatest amount of differentiation between the summer and fall populations (Figure 5c).

No peaks were detected for the FST and SFST tests when screening for genomic regions under selection among the summer‐run fish only. The FET test revealed a small peak on Chr9 where more than half of the SNPs are located in intergenic regions. Remaining SNPs are located on several different genes. Running FLK requires more than two populations for the test to build a rooting tree, therefore no FLK test was performed for the summer‐run fish.

In the last screening analysis among the fall‐run fish, no genomic regions under selection were detected using the FLK test. Similar to the first analyses using all populations and the second test between summer‐ and fall‐run fish, a small peak on Chr19 was detected for FET and FST test corresponding to the ladderlectin‐like gene. However, using the SFST test, the SNPs underlying the peak on Chr19 are located on a different gene called calcium‐independent phospholipase A2‐gamma like which promotes cellular membrane hydrolysis (Uniprot Q9NP80). Peaks on Chr13, Chr14, and Chr17 are shown using both the FST and SFST test. These peaks were also detected in the first and second analyses, and correspond to the same genomic regions, although only marginally detected when using fall‐run fish separately. The FST test also detected a small peak on Chr6 corresponding to SNPs located on a gene called baculoviral IAP repeat‐containing protein 6‐like. This protein has been seen to regulate cell death in mammalian cells (Bartke et al., 2004). The notable peak on Chr28 corresponding to the GREB1L/ROCK1 gene region that was detected by previous analyses was evident here as only a small peak using the SFST analysis. The SFST test also detected small peaks on Chr2, Chr6, Chr12, Chr18, and Chr21.

### 3.5 Genome–environment associations

Results from the four GEA analyses (RDA, AutoLM, LFMM, and BayPass) provided mixed findings on significant environmental features and candidate genes. All iterations of the RDA analyses using the Site, Migration, and Combined datasets returned no significant results. The full models and the axis models were non‐significant (*p* > .05) across all runs. Troubleshooting runs of this analysis suggested that even at a correlation threshold of 0.8, multicollinearity within the datasets presented an issue for implementing RDA tests. It is also likely that due to low variation in the environmental predictors across the seven populations, or the low number of populations tested, no signals were detected by the RDA approach (B.R. Forester 2020, personal communication, 2 Oct).

Analyses with AutoLM using the four environmental datasets showed no significant SNPs after multiple test correction for the Site dataset, only one SNP for the Combined dataset, two SNPs for the Migration dataset, and five SNPs for the Full dataset (with this dataset representing less stringent removal of correlated variables). From the Combined dataset, wind velocity at migration corridors (range) was associated with a single SNP on Chr20. From the Migration dataset, migration distance to the ocean was associated with one SNP on Chr2 and one SNP on Chr4. Lastly, from the Full dataset, annual precipitation (mean and range) at migration corridors were associated with a single SNP on Chr12, and diurnal temperature range (mean) at spawning sites was also associated with a single, but different SNP on Chr12. The maximum temperature of the warmest month (mean) at migration corridors was associated with a single SNP on Chr16, and wind velocity at the spawning site (mean) was associated with a single SNP on Chr22. Since AutoLM provided just a few candidate SNPs (total of eight SNPs) across all four environmental datasets tested and which were not consistent with other GEA results, we interpreted those results as false positives and did not include them in downstream analyses. Among our seven populations, which correspond to seven data points for each variable, we may not have represented the full extent of the variation in environmental predictors (Bogaerts‐Márquez et al., 2021). This loss of statistical power could explain why the RDA multivariate approach and the AutoLM univariate approach were not effective for our GEA analyses.

**FIGURE 6:**
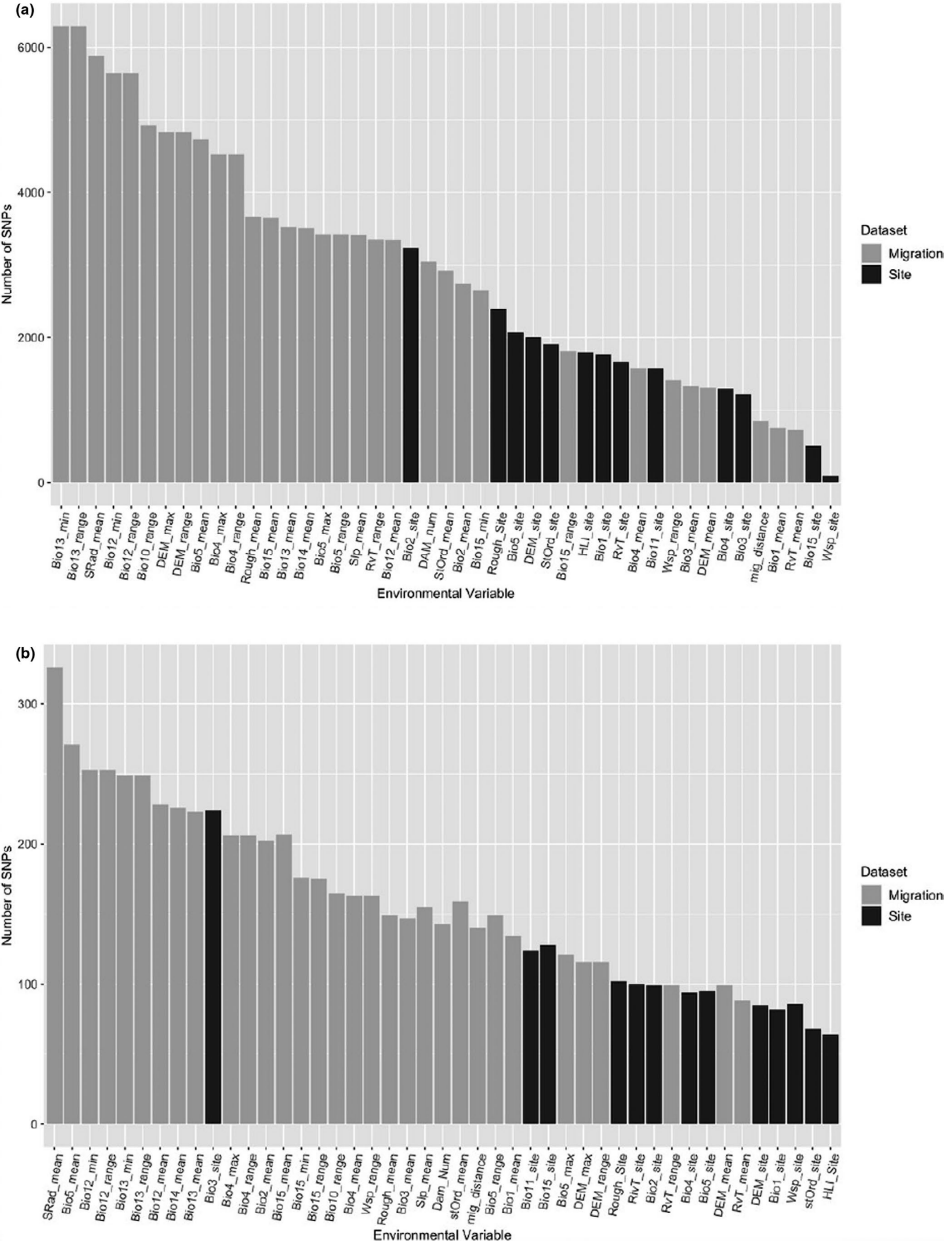
List of the environmental variables and their associated number of SNPs identified by the (a) LFMM analysis, and (b) BayPass analysis. Arranged from highest number of SNPs to lowest. Light gray shading indicates migration‐related variables; dark gray shading indicates site variables. Abbreviations for environmental variables correspond with those in Table 1

Results from the LFMM analysis returned a considerable number of significant SNPs. Overall, we found stronger associations (i.e., higher number of SNPs) with migration‐related variables rather than spawning site variables (Figure 6a). The top migration‐specific variables with the most associations were precipitation of the wettest month (minimum and range), precipitation (minimum and range), solar radiation, mean temperature of the warmest quarter (range), and elevation (maximum and range). There was a strong peak on Chr28 for elevation (maximum and range; Figure S4). For site‐specific variables, mean diurnal temperature range, terrain roughness, maximum temperature of the warmest month, stream order, and elevation, had the most associations (Figure S5). There was a strong peak on Chr28 for all these top site‐specific variables. Several additional variables showed a strong peak on Chr28; for site‐specific variables, these were mean temperature of the coldest quarter, annual mean temperature, temperature seasonality (standard deviation ×100), August water temperature, and Heat Load Index. For migration‐specific variables, these were elevation (mean) and August water temperature (mean; Figure S6). Filtering for significant SNPs and across all four runs of LFMM, yielded 36,675 SNPs, of which most were associated with Chr1 (6.28%), Chr28 (5.98%), Chr6 (5.49%), Chr2 (4.87%), and Chr8 (4.49%).

For the four BayPass analyses, there was no one region of the genome in which there was a cluster of significant SNP positions. Overall, there were a higher number of SNPs identified as significant (BF > 20) with migration‐related variables compared with spawning site variables (Figure 6b). The top migration variables with the most associations were solar radiation, the maximum temperature of the warmest month, the annual precipitation, and the precipitation of the wettest month. For the site‐specific variables, the top variables with the most associations across the genome were isothermality, precipitation seasonality, mean temperature of the coldest quarter, and terrain roughness.

Multiple GEA approaches were used in this study in order to compare across analysis types (i.e., Bayesian vs. univariate and multivariate frequentist methods) and to mitigate false positives and negatives. Of the four GEA tests performed, only BayPass and LFMM provided a set of putatively adaptive SNPs with significant associations with environmental variables. We were unsuccessful in using redundancy analysis despite multiple attempts.

**FIGURE 7:**
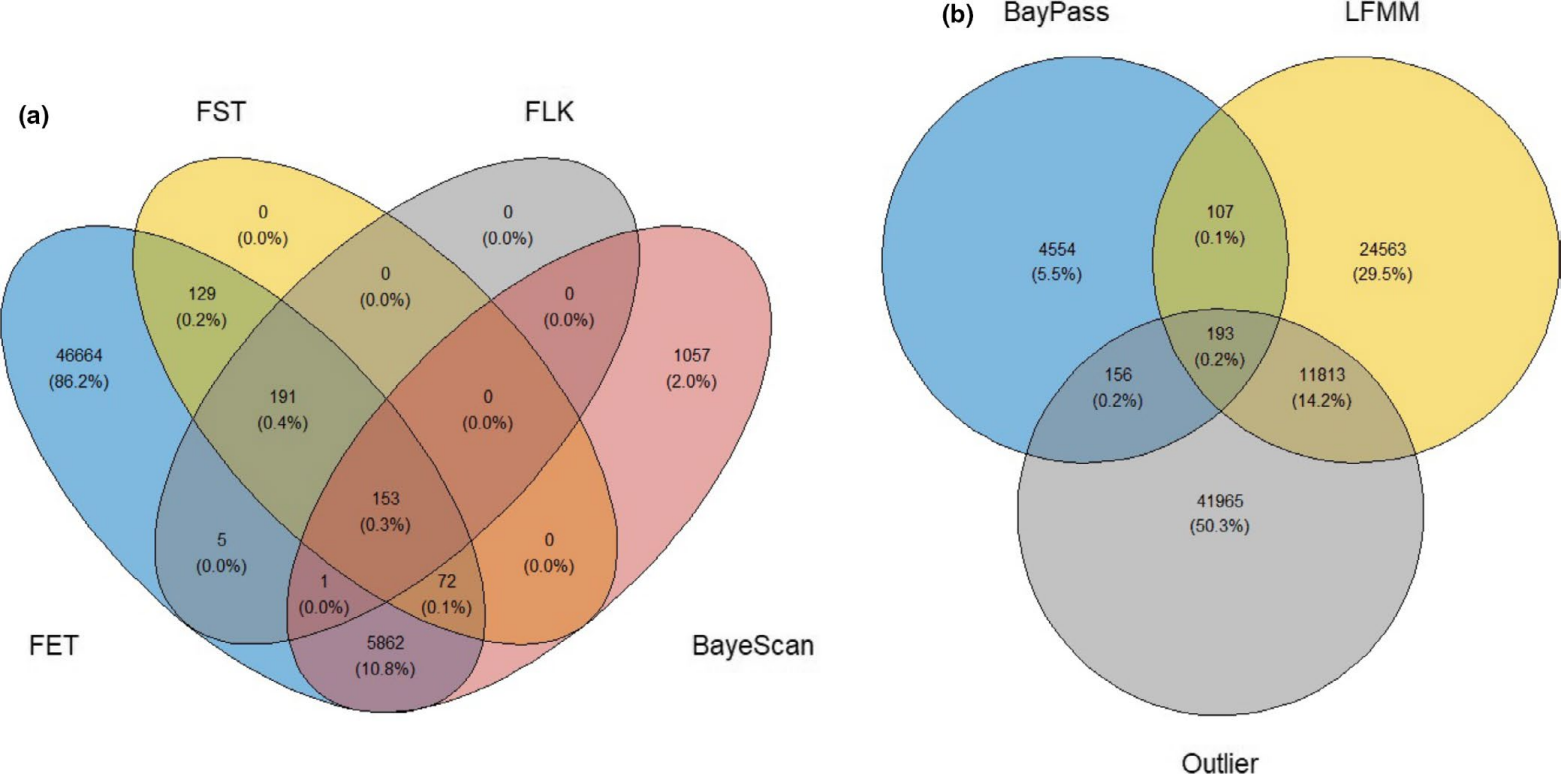
Venn diagrams showing the number of significant SNPs identified by (a) the four outlier tests, and from (b) BayPass, LFMM2, and the outlier tests combined

Filtering for SNPs in common between at least one BayPass test and one univariate test (LFMM) yielded a set of 300 candidate SNPs (Figure 7). Of those 300 candidate SNPs, 193 SNPs were in common with at least one outlier test (FST, SFST, FET, or FLK). Most of the 300 SNPs in common between BayPass and LFMM were on Chr1 (10.7%), Chr28 (10.36%), and Chr2 (8.0%). There were no significant GO terms associated with any of the 300 candidate SNPs. Similarly, there were no significant GO terms associated with the set of SNPs in common between at least one outlier test and one GEA test.

## 4 DISCUSSION

In this study, we examined seven populations of Chinook salmon (interior ocean‐type lineage) from the Columbia River basin to understand the relationship between landscape and environmental conditions driving selection in this species. We assessed the neutral population genetic structure among populations to aid in discerning signatures of underlying population structure and genomic signatures that may be correlated to environmental variables. Through different combinations of environmental datasets and GEA methods, we show that environmental variation experienced throughout the shared migration corridor imposed a greater selective pressure on Chinook salmon than conditions at spawning sites. The genomic regions identified by GEA analyses were generally independent (14% of SNPs overlapped between the LFMM and outlier tests) from those regions identified by outlier tests that did not incorporate environmental variables.

Population genetic structure based on putatively neutral markers identified two main clusters corresponding to summer‐run and fall‐run populations. This level of neutral divergence is greater than determined with previous studies of Chinook salmon in the interior Columbia River that found no significant divergence between summer‐ and fall‐run fish in fine‐scale population structure analyses from multiple marker types (Narum et al., 2008; Waples et al., 2004). While we still estimated low FST values between summer‐ and fall‐run populations (mean of 0.006), the vast number of neutral markers in this study likely provided improved power to distinguish these populations over previous studies. However, the outlier tests with all SNPs across the genome confirmed that the main driver of genetic divergence based on run‐type is due to a region on Chr28 (GREB1L/ROCK1; Figure 5c) that has been identified previously in Chinook salmon (Narum et al., 2018; Prince et al., 2017; Thompson et al., 2019, 2020; Waples & Lindley, 2018; Willis et al., 2021).

Beyond the neutral population genetic structure of a species, environmental and topographic variation across a species’ native landscape can generate local selective pressures that contribute to local adaptive divergence (Hecht et al., 2015). Our cluster analyses based on the environmental conditions indicated that locations that were more environmentally similar were occupied by populations with similar migration timing, and summer‐run populations cluster separately from those migrating in the fall. This suggests that there may be an interplay between the selective pressures of migration timing and the local environmental conditions during migration—for example, high or low river flows, water temperature–that ultimately contribute to the overall genomic signatures observed in Chinook salmon.

Among topographic features, terrain roughness (ruggedness) was one of the top variables associated with adaptive genetic variation at spawning sites and identified by two GEA methods. Terrain ruggedness provides a measure of topographic heterogeneity. Specifically, it is a measure of elevation differences between a grid cell and its neighboring cells (Riley et al., 1999) and reflects stream gradient in freshwater ecosystems. Elevation was another topographic feature that had strong associations with adaptive genetic variation at both migration corridors and spawning sites. Higher elevation habitat tends to be cooler, whereas lower elevation habitat tends to be warmer. Warmer water environments at lower elevations could create strong selective pressures on Chinook salmon (see below for further discussion of water temperature). Stream gradient and elevation are important features consistent with migratory timing as fall‐run Chinook salmon primarily spawn in low gradient mainstem sites versus summer‐run fish that spawn in tributary locations (Myers et al., 1998).

The long, up‐stream migration distance to return to spawning grounds by salmonids is an energetically costly process (Bowerman et al., 2017; Brett, 1995; Crossin et al., 2004; Mesa & Magie, 2006). Previous genomic work has identified migration distance as an important variable for adaptive genetic variation in Chinook salmon (Hecht et al., 2015) and Steelhead (Micheletti et al., 2018). Similarly, here we identified migration distance through one GEA analysis (BayPass) as an important environmental variable. This measure reflects both the distance that juvenile salmonids travel to reach the ocean and the distance adults travel to return to spawning sites (Healey, 1991; Willis et al., 2021), impacting fish during multiple life history stages, and could explain why migration distance may impose a strong selective pressure on Chinook salmon. Longer migration distance also tends to be correlated with increased encounters with dams (Mesa & Magie, 2006). Interestingly, although there were candidate SNPs associated with the number of dams along migration corridors, this was not one of the top variables identified by the GEA analyses. The number of dams encountered was a significant variable in Steelhead returning to the Columbia River basin (Micheletti et al., 2018), however, many of the Steelhead populations used for that study were located in the upper Salmon River basin. These fish have several more dams to cross before reaching their spawning grounds compared with most of the Chinook salmon populations in the current study.

For temperature‐related variables, maximum temperature of the warmest month was consistently identified as a variable with strong associations with adaptive genetic variation at both migration corridors and spawning sites. Temperature is known to have important potential contributions to local adaptation in various salmonids and can influence various phenotypic changes across the life stages of fish, such as migration and spawning timing, growth, fecundity, among others (Crozier & Hutchings, 2014; Muñoz et al., 2015). Therefore, it is not surprising that we found temperature to be an important environmental variable at both migration and spawning sites. Water temperature, represented as 20‐year mean of August water temperatures, was also a relatively top variable in our GEA analyses, and there is evidence in the literature for the important role that water temperature plays for the life history of salmonids (Crozier & Hutchings, 2014). For example, water temperature can influence swim speed of fish. At a determined optimum of 16°C, the swim speed of migratory Chinook salmon was found to increase when water temperature was above the optimum, and to decrease when the temperature was below the optimum (Salinger & Anderson, 2006).

Precipitation‐related variables pertaining to migration corridors were consistently identified as top variables by GEA tests. Precipitation‐related variables have previously been signaled as drivers of adaptive genetic variation in salmonids (Bourret et al., 2013; Hecht et al., 2015; Matala et al., 2014; Olsen et al., 2011). Our results add more evidence showing that precipitation‐related variables are important drivers of environmental selection; yet, we also show here that the effect of precipitation is not identical between migration and spawning sites. Among precipitation variables, we found that annual precipitation, precipitation seasonality, and precipitation of the wettest month were strongly associated with adaptive genetic variation at migration corridors. Interestingly, only precipitation seasonality was an important variable for spawning sites. It is possible that precipitation conditions at migration corridors that influence river flow levels are crucial for juveniles that are beginning their migration to sea, or adults returning to spawn (e.g., Hecht et al., 2015; Keefer et al., 2017), since river flow can be a determinant of migration speed (Salinger & Anderson, 2006).

There were more SNPs in common between the LFMM test and the outlier tests (14%) compared with the BayPass tests and the outlier tests (0.2%; Figure 7). Several of the migration‐specific and spawning site‐specific environmental variables with associations to adaptive genetic variation exhibited strong peaks on Chr28, especially from the LFMM test (Figures A4, A5 and A6). These peaks correspond to the GREB1L/ROCK1 region that has been associated with adult migration timing (Narum et al., 2018; Prince et al., 2017; Thompson et al., 2019, 2020; Waples & Lindley, 2018; Willis et al., 2021). This provides further evidence of the likely interplay between environmental selection and adult migration timing in Chinook salmon.

Across neutral outlier tests and GEA tests, we identified 193 candidate SNPs in common for adaptive selection but did not have any significant GO enrichment based on GO analysis. Given that studies investigating environmental selection in Chinook salmon reported that 5.8–21.8% of genomic variation may be driven by environmental conditions (Hecht et al., 2015), and that we observed candidate SNPs in common between both outlier tests and GEA tests, this may indicate that these genomic signals represent adaptive variation in the genome. We infer that environmental conditions across Chinook salmon habitats are strong drivers of selection, and that migration‐related variables may be imposing stronger selection pressures on Chinook salmon compared with spawning sites, similarly to what has been shown in anadromous steelhead in the Columbia River Basin (Micheletti et al., 2018).

## 5 CONCLUSIONS

Accounting for local adaptation has become increasingly important in contemporary conservation management strategies because differences in local adaptation often indicate responses to different environmental threats (Muñoz et al., 2015; Supple & Shapiro, 2018). Understanding which environmental variables drive local genetic variation in Chinook salmon will allow better conservation management of distinct populations and ensure population sustainability for future generations (Funk et al., 2012; Waples & Lindley, 2018). At migration corridors, we found that stream gradient and elevation are important topographic features with important roles in migration timing and may generate local selective pressures. Migration distance and water temperature, which introduce energetic costs and influence the optimal swim speed of fish, respectively, are also significant environmental features associated with adaptive divergence. We identified maximum air temperature and precipitation conditions as two climate variables that may also drive environmental selection, likely due to their influence on various aspects of the life history of fish such as migration and spawning timing. Our research suggests that environmental conditions at migration corridors may be more influential on Chinook salmon genetic variation compared with spawning site environmental conditions. Previous studies have suggested that both neutral and adaptive variation should be considered when delineating conservation units, as basing them solely on overall genetic differentiation might fail to preserve evolutionarily important variation (Funk et al., 2012; Prince et al., 2017; Waples & Lindley, 2018). Our work adds to the growing body of landscape genomics that integrates both spawning/juvenile rearing sites and migration pathways (Micheletti et al., 2018). This approach provides a more complete assessment of environmental pressures as they relate to the life history of a species, as it takes into account the various habitats that a migratory species occupies throughout its life.

## CONFLICT OF INTEREST

The authors declare no competing interests.

## AUTHOR CONTRIBUTIONS

**Yara A. Alshwairikh:** Data curation (equal); Formal analysis (equal); Methodology (equal); Software (equal); Visualization (equal); Writing‐original draft (equal); Writing‐review & editing (equal). **Shayla L. Kroeze:** Visualization (equal); Writing‐original draft (equal); Writing‐review & editing (equal). **Jenny Olsson:** Data curation (equal); Formal analysis (equal); Software (equal); Visualization (equal); Writing‐original draft (equal); Writing‐review & editing (equal). **Steve A. Stephens‐Cardenas:** Formal analysis (equal); Software (equal); Visualization (equal); Writing‐original draft (equal); Writing‐review & editing (equal). **William L. Swain:** Visualization (equal); Writing‐original draft (equal); Writing‐review & editing (equal). **Lisette Waits:** Conceptualization (equal); Investigation (equal); Methodology (equal); Writing‐review & editing (equal). **Rebekah L. Horn:** Conceptualization (equal); Data curation (equal); Formal analysis (equal); Investigation (equal); Methodology (equal); Software (equal); Visualization (equal); Writing‐review & editing (equal). **Shawn Narum:** Conceptualization (equal); Funding acquisition (equal); Investigation (equal); Methodology (equal); Writing‐review & editing (equal). **Travis Seaborn:** Conceptualization (equal); Data curation (equal); Formal analysis (equal); Funding acquisition (equal); Investigation (equal); Methodology (equal); Software (equal); Writing‐review & editing (equal).

## Supporting information

Fig S1

Fig S2

Fig S3

Fig S4

Fig S5

Fig S6

Table S1‐S2

Supplementary Material

## Data Availability

Raw sequence reads are available on NCBI under BioProject PRJNA763859, accession numbers SAMN21454079, SAMN21454080, SAMN21454081, SAMN21454082. Additional sequence data used in this study were previously published under BioProject PRJNA579979. The allele frequency matrix used in GEA analyses, and environmental data including a correlation matrix are available on Dryad: https://doi.org/10.5061/dryad.prr4xgxmn. R code and scripts are available on the Github repository: https://github.com/YaraAlshw/LG_Chinook.
