# Supplementary figures and images for: Influence of environmental conditions at spawning sites and migration routes on adaptive variation and population connectivity in Chinook salmon
Source: Ecol Evol. 2021 Nov 16;11(23):16890–908. doi: 10.1002/ece3.8324 (PMC8668735; doi:10.1002/ece3.8324)

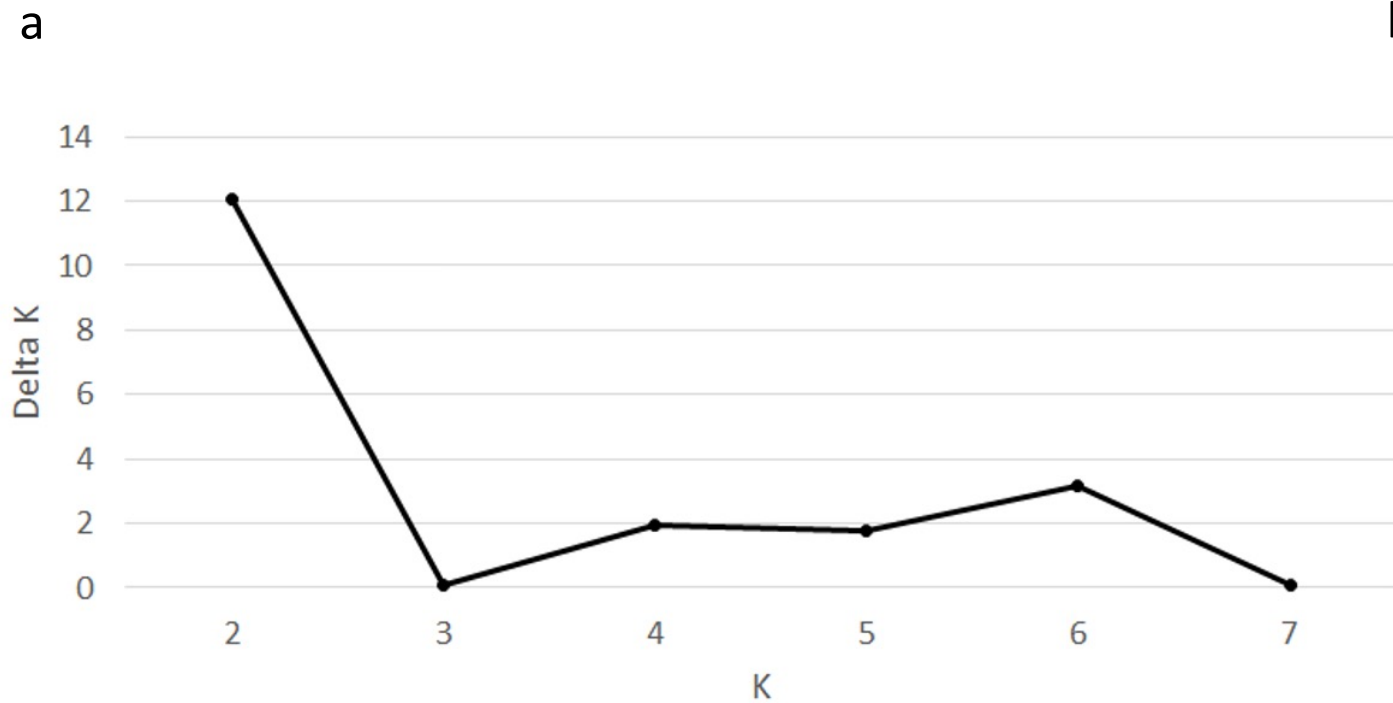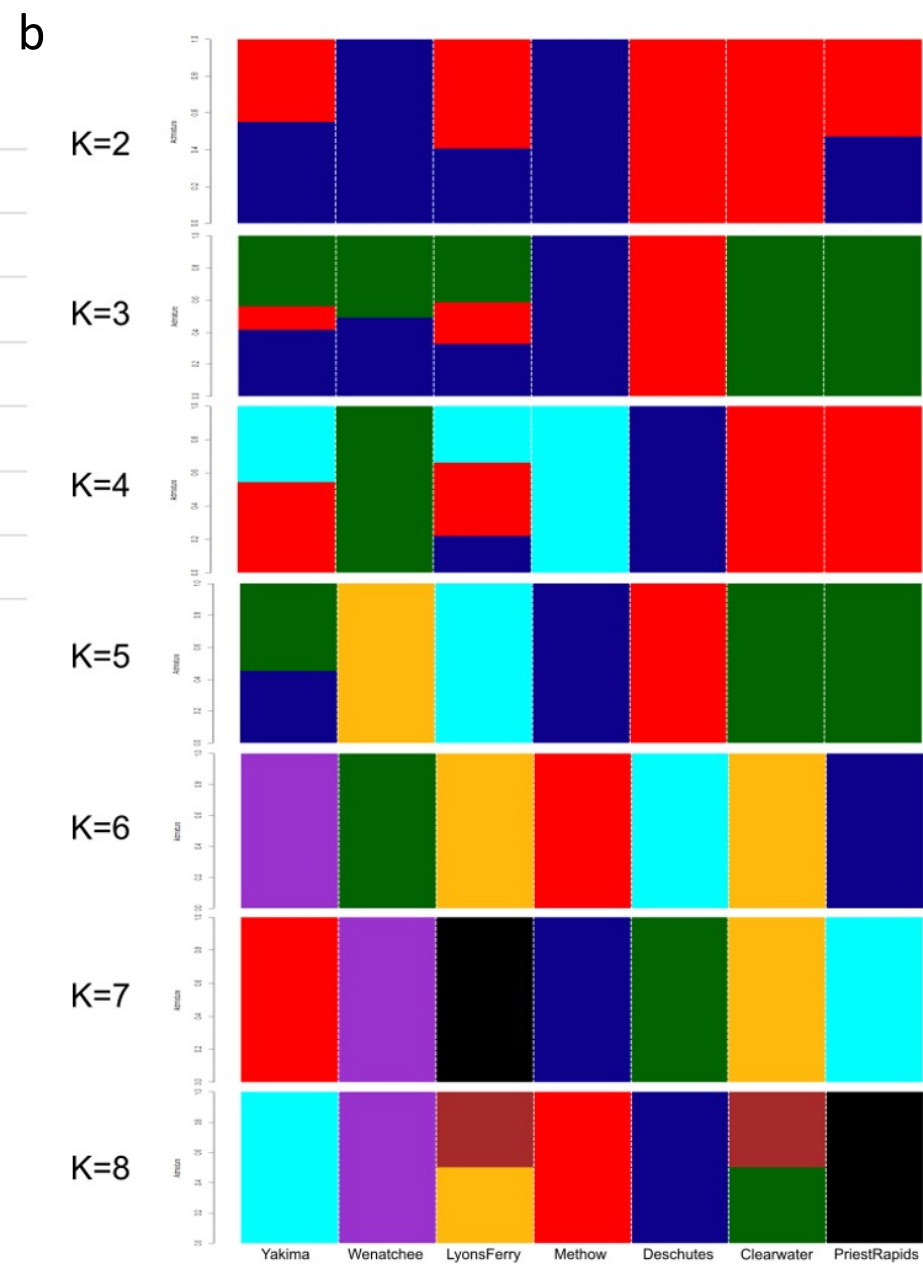

Supplement: Supplementary file 1 — Fig S1 [file ECE3-11-16890-s007.pdf]

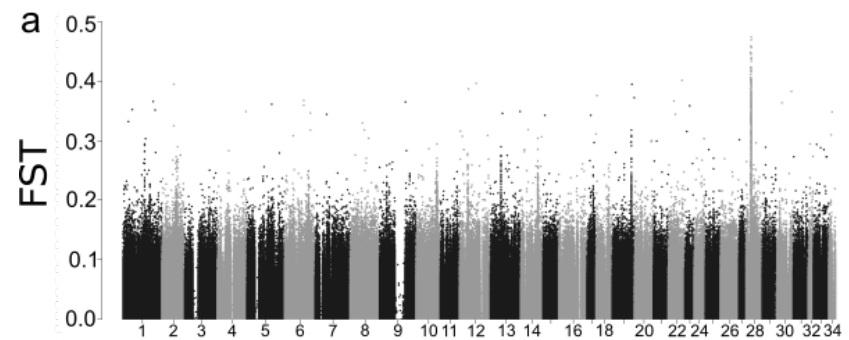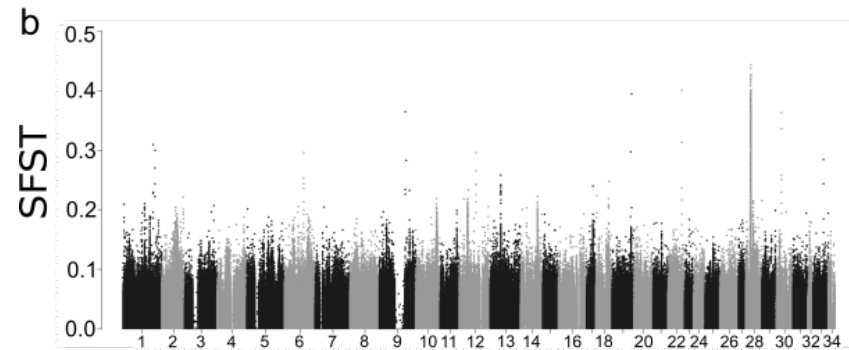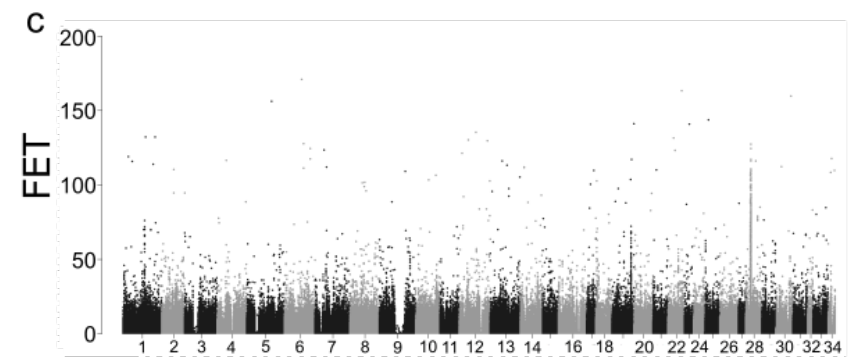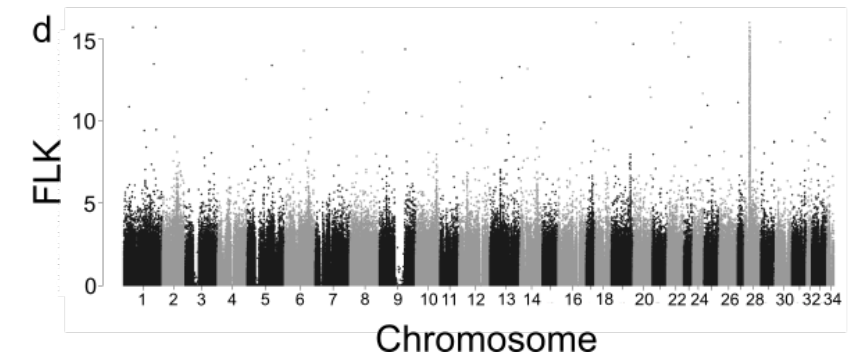

Supplement: Supplementary file 2 — Fig S2 [file ECE3-11-16890-s006.pdf]

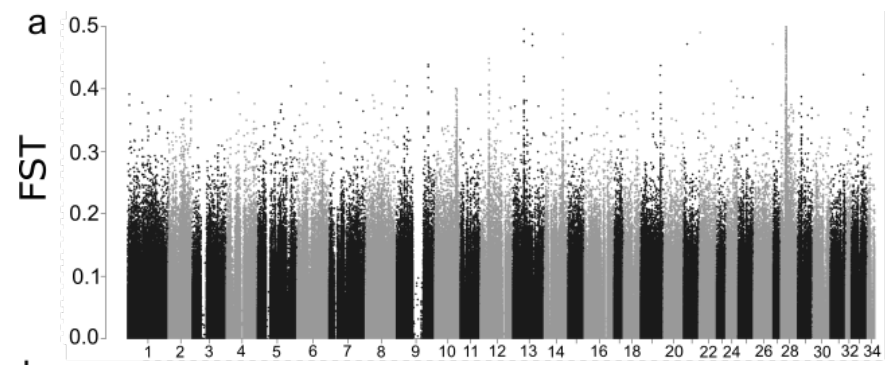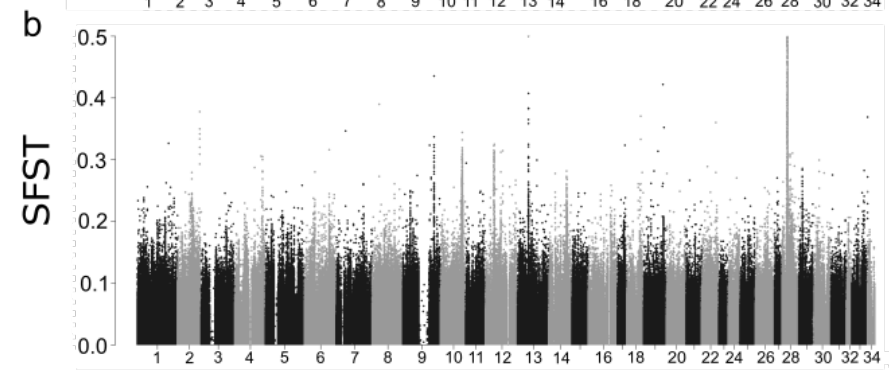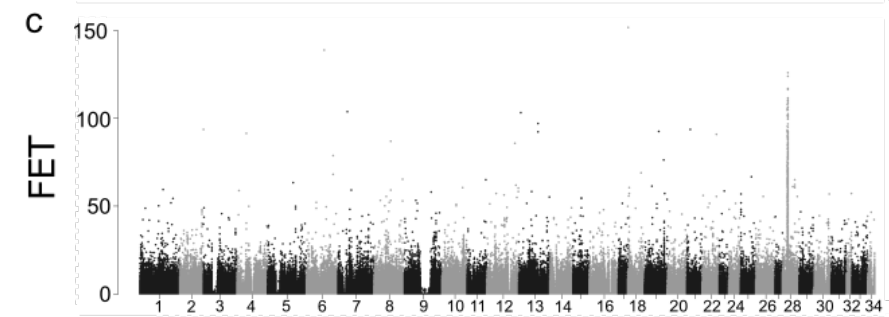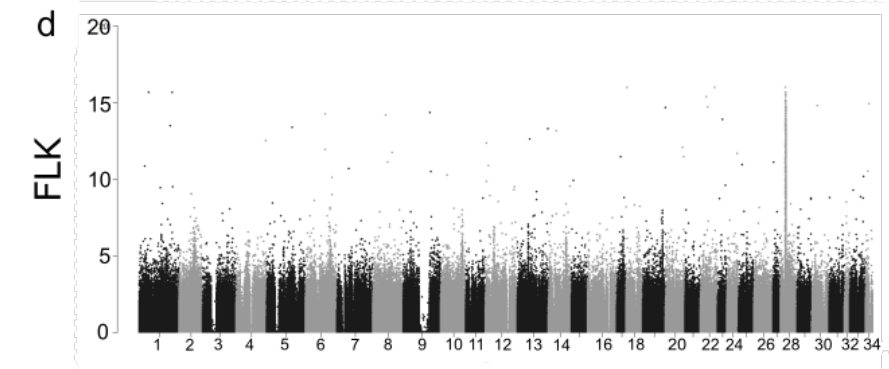

Chromosome

Supplement: Supplementary file 3 — Fig S3 [file ECE3-11-16890-s001.pdf]

-log<sub>10</sub>(pval)

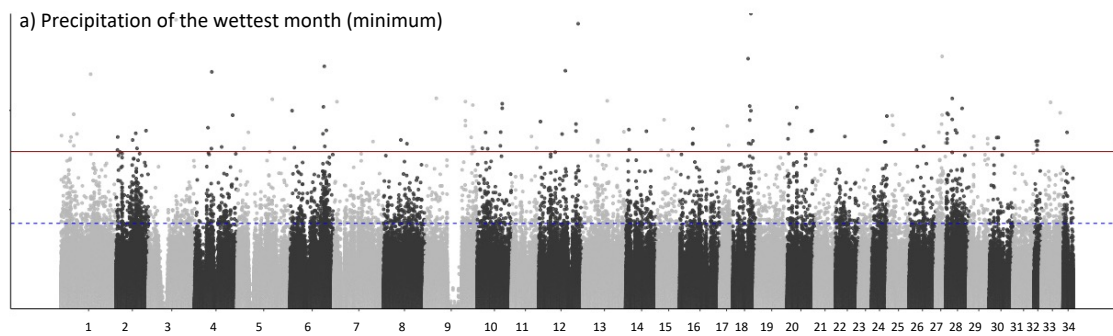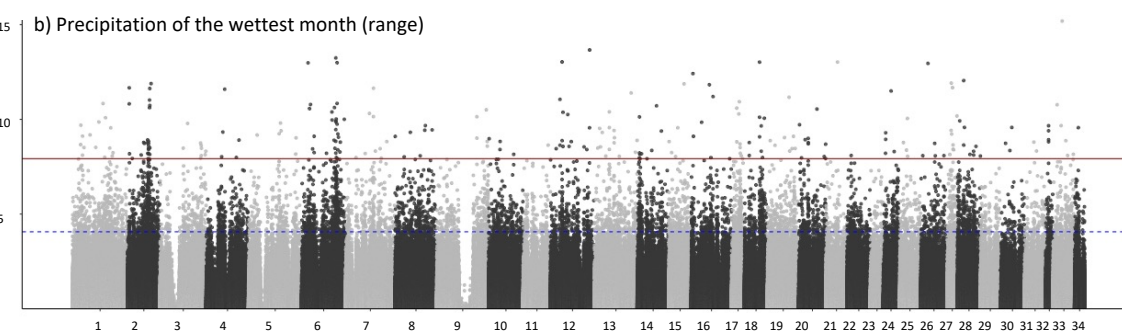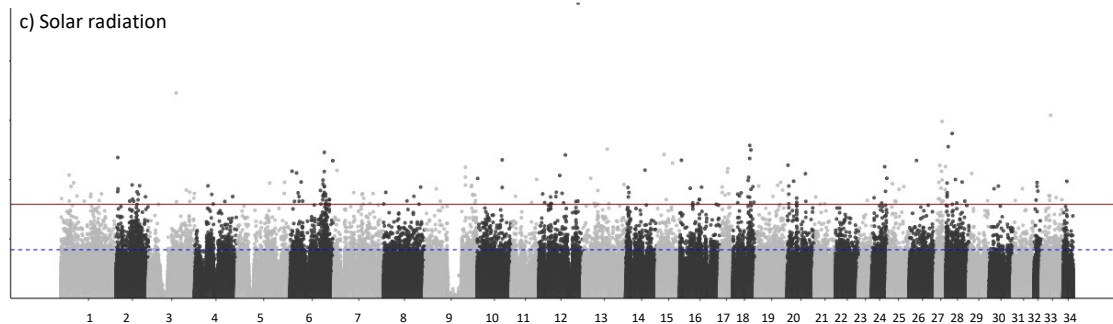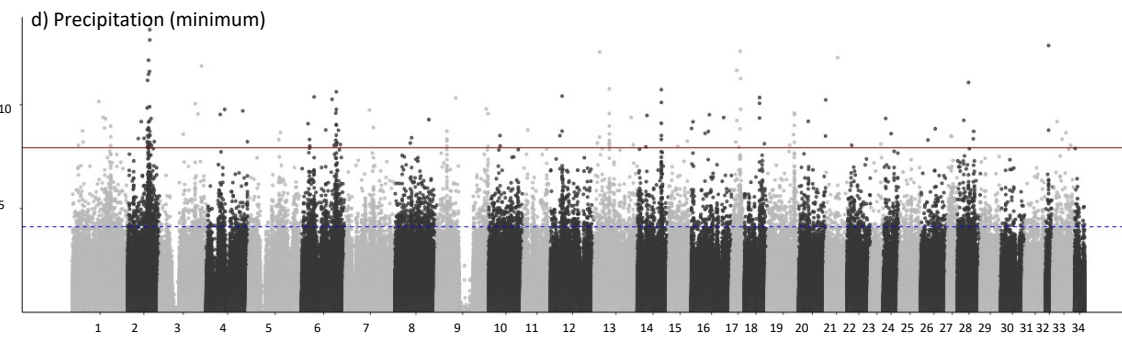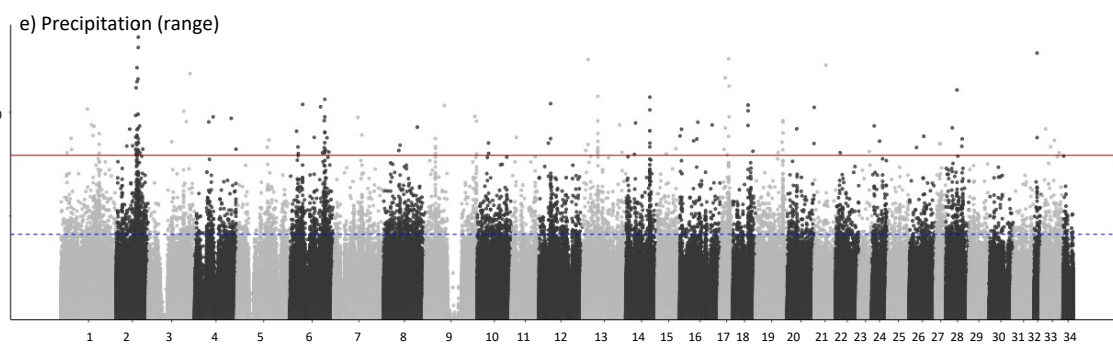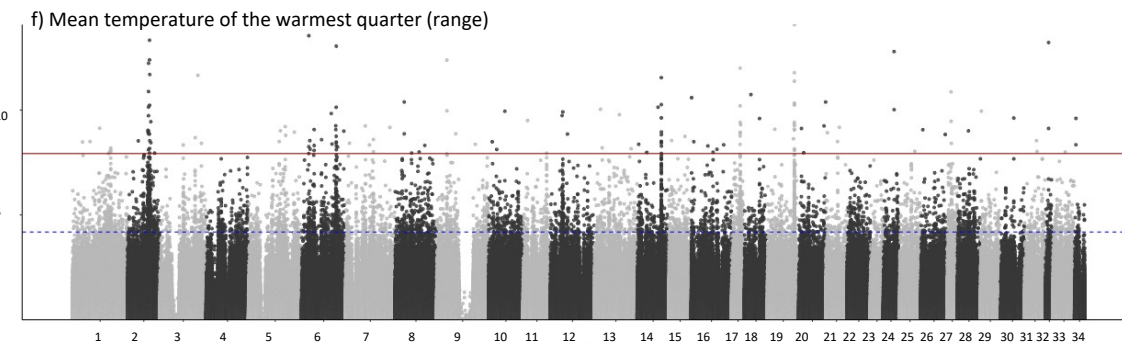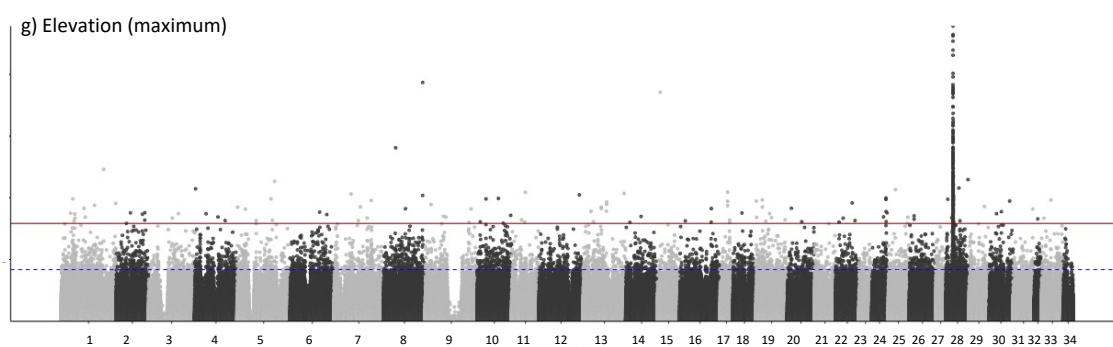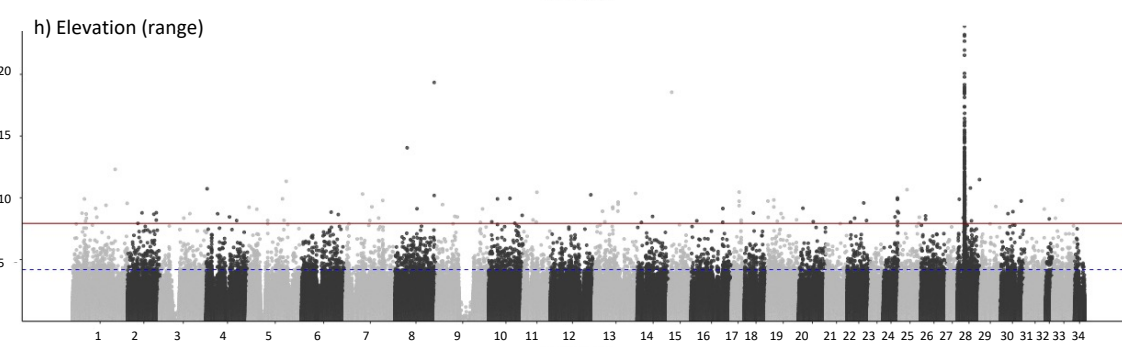

chromosome

Supplement: Supplementary file 4 — Fig S4 [file ECE3-11-16890-s008.pdf]

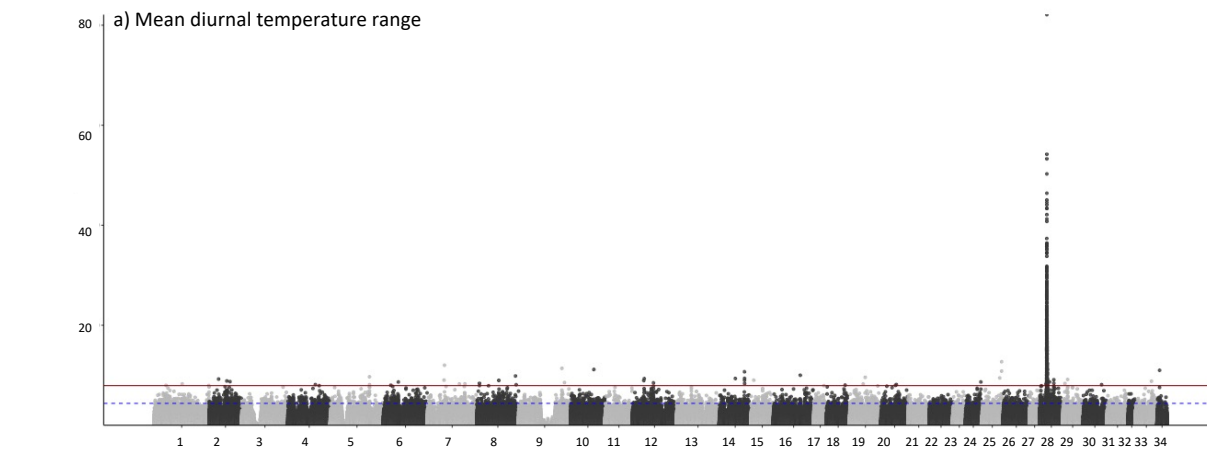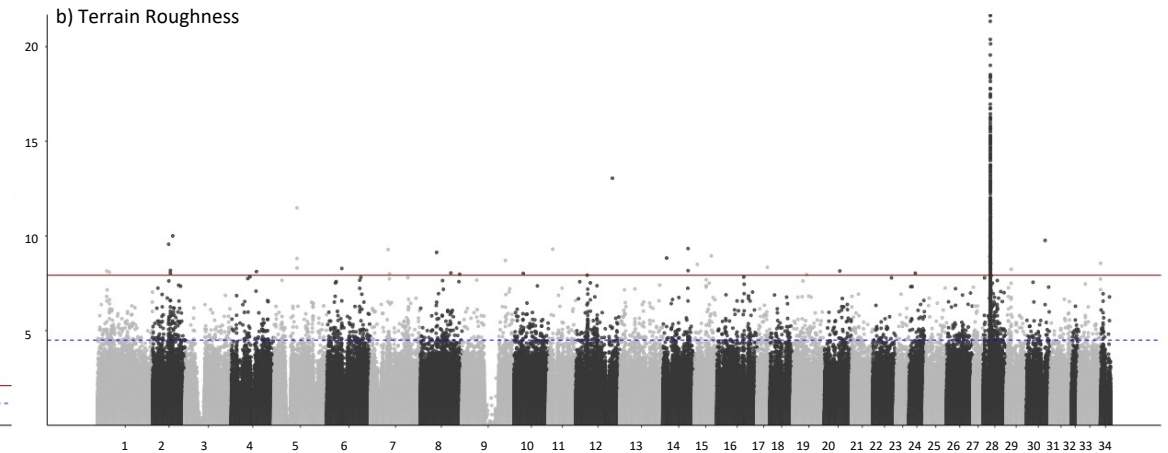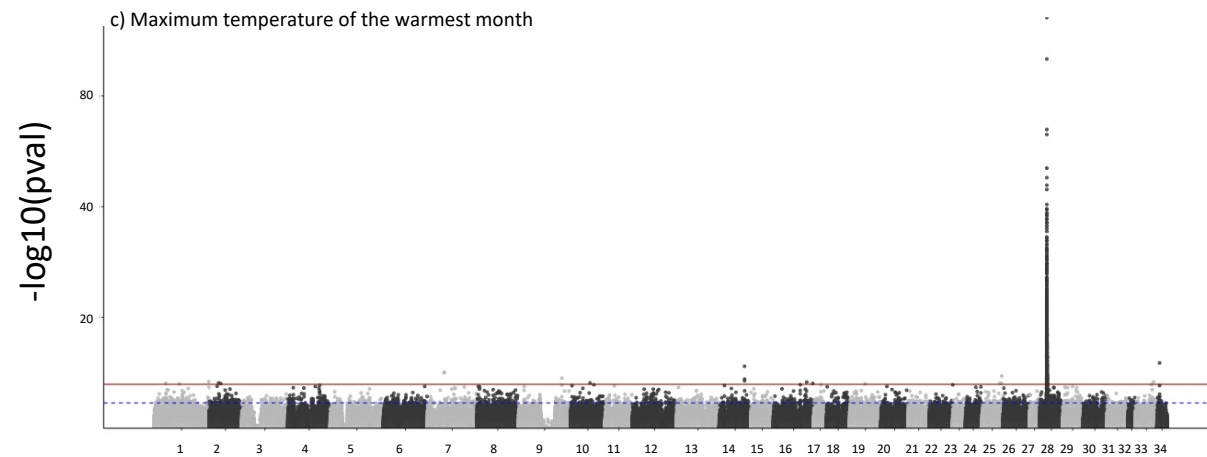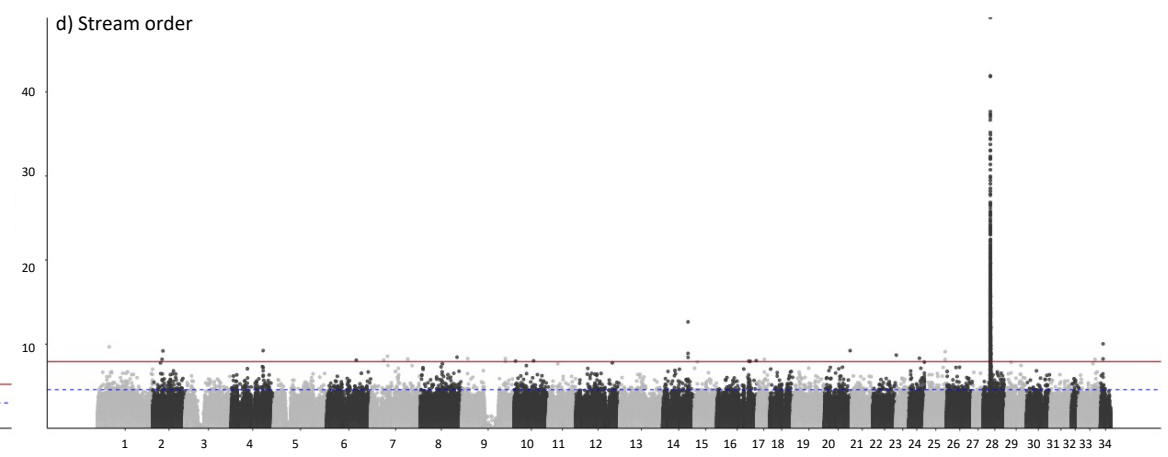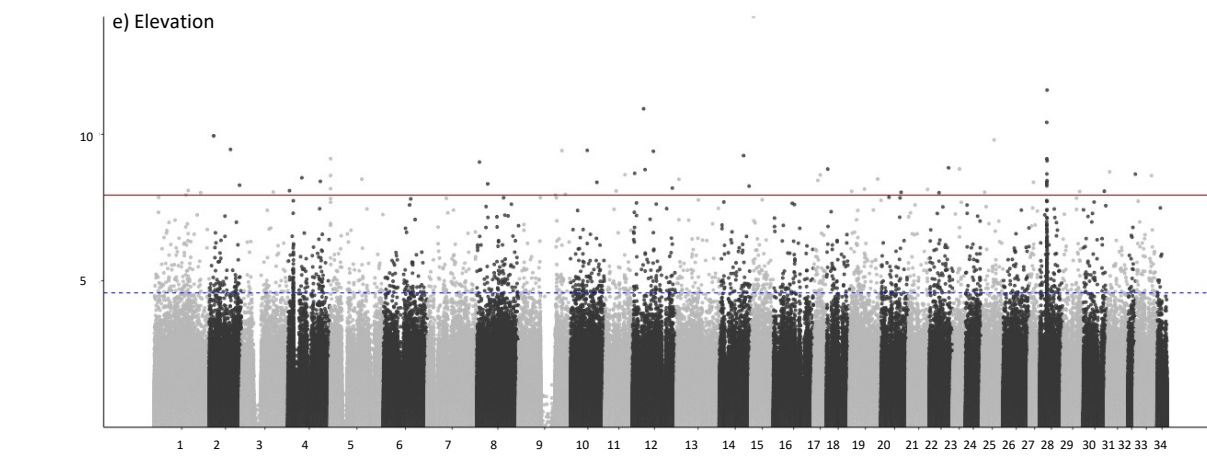

chromosome

Supplement: Supplementary file 5 — Fig S5 [file ECE3-11-16890-s003.pdf]

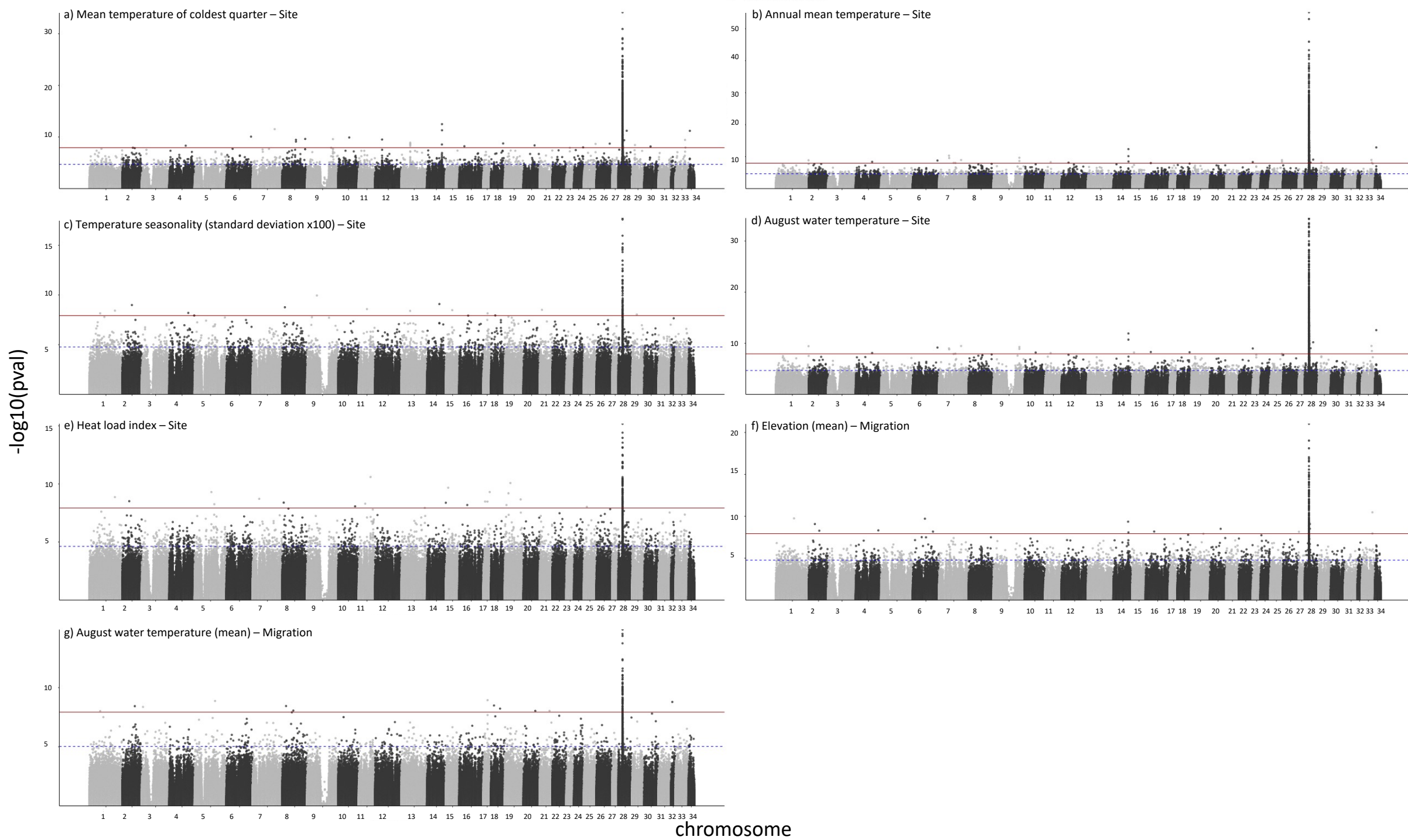

Supplement: Supplementary file 6 — Fig S6 [file ECE3-11-16890-s004.pdf]
