# Supplementary material for: Influence of environmental conditions at spawning sites and migration routes on adaptive variation and population connectivity in Chinook salmon
Source: Ecol Evol. 2021 Nov 16;11(23):16890–908. doi: 10.1002/ece3.8324 (PMC8668735; doi:10.1002/ece3.8324)
Supplement: Supplementary file 7 — Table S1‐S2 [file ECE3-11-16890-s002.docx]

**Table S1.** Pairwise FST comparisons for all populations with the lower and upper confidence intervals (CI) reported and grouped by migration return season, summer (su) and fall (fa).

| **Season** | **Pairwise Comparison** | **FST** | **Lower CI** | **Upper CI** |
| --- | --- | --- | --- | --- |
| fa-fa | LYakima-PriestRapids | -0.006 | -0.007 | -0.006 |
| fa-fa | LYakima-LyonsFerry | -0.001 | -0.002 | -0.002 |
| fa-fa | Clearwater-PriestRapids | 0.002 | 0.001 | 0.001 |
| fa-fa | LyonsFerry-PriestRapids | 0.002 | 0.002 | 0.002 |
| fa-fa | Clearwater-LyonsFerry | 0.002 | 0.002 | 0.002 |
| fa-fa | Clearwater-LYakima | 0.003 | 0.003 | 0.003 |
| fa-fa | Deschutes-LyonsFerry | 0.008 | 0.007 | 0.008 |
| fa-fa | Deschutes-LYakima | 0.009 | 0.008 | 0.008 |
| fa-fa | Clearwater-Deschutes | 0.011 | 0.009 | 0.010 |
| fa-fa | Deschutes-PriestRapids | 0.012 | 0.010 | 0.011 |
| su-fa | PriestRapids-Wenatchee | -0.002 | -0.002 | -0.002 |
| su-fa | LYakima-Wenatchee | -0.001 | -0.001 | -0.001 |
| su-fa | LYakima-Methow | 0.001 | 0.001 | 0.001 |
| su-fa | PriestRapids-Methow | 0.008 | 0.008 | 0.008 |
| su-fa | Clearwater-Wenatchee | 0.011 | 0.011 | 0.011 |
| su-fa | LyonsFerry-Methow | 0.011 | 0.011 | 0.011 |
| su-fa | LyonsFerry-Wenatchee | 0.012 | 0.011 | 0.012 |
| su-fa | Clearwater-Methow | 0.021 | 0.020 | 0.020 |
| su-fa | Deschutes-Wenatchee | 0.024 | 0.022 | 0.023 |
| su-fa | Deschutes-Methow | 0.027 | 0.025 | 0.026 |
| su-su | Wenatchee-Methow | 0.004 | 0.004 | 0.004 |

**Table S2.** Pairwise comparisons for overlap of migration routes for all seven populations of Chinook salmon. Overlap percent as estimated in Barraclough & Vogler (2000)

| **Migration Paths** | | **Shared Distance (Km)** | **% Overlap to smallest river** |
| --- | --- | --- | --- |
| **First River** | **Second River** |  |  |
| Methow R summer-run | Wenatchee R summer-run | 889.6 | 93.4 |
|  | Upper Deschutes River fall-run | 351.9 | 79.9 |
|  | Lower Yakima R fall-run | 636.5 | 92.8 |
|  | Priest Rapids fall-run | 759.1 | 100 |
|  | Clearwater River fall-run | 614.3 | 67.5 |
|  | Lyons Ferry weir fall-run | 614.3 | 83.6 |
| Wenatchee R summer-run | Upper Deschutes River fall-run | 351.9 | 79.9 |
|  | Lower Yakima R fall-run | 636.5 | 92.8 |
|  | Priest Rapids fall-run | 759.1 | 100 |
|  | Clearwater River fall-run | 614.3 | 67.5 |
|  | Lyons Ferry weir fall-run | 614.3 | 83.6 |
| Upper Deschutes River fall-run | Lower Yakima R fall-run | 354.7 | 80.6 |
|  | Priest Rapids fall-run | 354.7 | 80.6 |
|  | Clearwater River fall-run | 354.7 | 80.6 |
|  | Lyons Ferry weir fall-run | 354.7 | 80.6 |
| Lower Yakima R fall-run | Priest Rapids fall-run | 636.5 | 92.8 |
|  | Clearwater River fall-run | 614.3 | 89.6 |
|  | Lyons Ferry weir fall-run | 614.3 | 89.6 |
| Priest Rapids fall-run | Clearwater River fall-run | 614.3 | 80.9 |
|  | Lyons Ferry weir fall-run | 614.3 | 83.6 |
| Clearwater River fall-run | Lyons Ferry weir fall-run | 735.1 | 100 |
