# Supplementary material for: Influence of environmental conditions at spawning sites and migration routes on adaptive variation and population connectivity in Chinook salmon
Source: Ecol Evol. 2021 Nov 16;11(23):16890–908. doi: 10.1002/ece3.8324 (PMC8668735; doi:10.1002/ece3.8324)
Supplement: Supplementary file 8 — Supplementary Material [file ECE3-11-16890-s005.docx]

**Figure S1.** a) The results from NGSadmix on the set of putatively neutral SNPs on a K ranging from 2 to 8 using the Evanno method (Evanno et al. 2005), and in b) the ancestry plot for the seven populations of Chinook salmon. At a K=2, the populations are generally split by summer- and fall-run populations, however, there was summer-run ancestry in the Yakima River, Lyons Ferry, and Priest Rapids populations. At K=7, sample locations do not show any mixed ancestry (K=7), or some slight mixed ancestry between the Lyons Ferry and Clearwater River populations (K=8)

**Figure S2**. Manhattan plots for the analyses detecting significant genomic regions among all populations of Chinook salmon using the a) FST test, b) sliding window FST (SFST) test, c) Fisher’s exact test (FET), and d) extended Lewontin and Krakauer (FLK) test.

**Figure S3**. Manhattan plots for the analyses detecting significant genomic regions among summer versus fall populations of Chinook salmon using the a) FST test, b) sliding window FST (SFST) test, c) Fisher’s exact test (FET), and d) extended Lewontin and Krakauer (FLK) test.

**Figure S4**. Manhattan plots for the LFMM analysis showing associations between individual environmental variables and SNPs. Represented here are the top eight migration-specific environmental variables, a) minimum precipitation of the wettest month (minimum), b) precipitation of the wettest month (range), c) solar radiation, d) precipitation (minimum), e) precipitation (range), f) mean temperature of the warmest quarter (range), g) elevation (maximum), and h) elevation (range). Red horizontal line represents the Bonferroni correction, and the blue dashed horizontal line represents the BH correction threshold.

**Figure S5.** Manhattan plots for the LFMM analysis showing associations between individual environmental variables and SNPs. Represented here are the top five site-specific environmental variables, a) mean diurnal range temperature, b) terrain roughness, c) maximum temperature of the warmest month, d) stream order, and e) elevation. Red horizontal line represents the Bonferroni correction, and the blue dashed horizontal line represents the BH correction threshold.

**Figure S6**. Manhattan plots for the LFMM analysis showing associations between individual environmental variables and SNPs. Represented here are the additional Manhattan plots showing a strong peak on Chr28 beyond the top five site-specific environmental variables, and the top eight migration-specific variables. The site-specific variables are a) mean temperature of the coldest quarter, b) annual mean temperature, c) temperature seasonality (standard deviation x100), d) august water temperature, and e) heat load index. The migration-specific variables are f) elevation, and g) August water temperature (mean). Red horizontal line represents the Bonferroni correction, and the blue dashed horizontal line represents the BH correction threshold.

References

Evanno G., Regnaut S., Goudet J. (2005). Detecting the number of clusters of individuals using the software structure: a simulation study. *Molecular Ecology, 14(8), 2611-2620.*
